# Unlocking cellular barriers: silica nanoparticles and fullerenol conjugated cell-penetrating agents for enhanced intracellular drug delivery

**DOI:** 10.3389/fbioe.2023.1184973

**Published:** 2023-05-09

**Authors:** Eduardo Ravelo-Nieto, Javier Cifuentes, Paola Ruiz Puentes, Laura Rueda-Gensini, Valentina Quezada, Carlos Ostos, Carolina Muñoz-Camargo, Luis H. Reyes, Alvaro Duarte-Ruiz, Juan C. Cruz

**Affiliations:** ^1^ Department of Chemistry, Universidad Nacional de Colombia, Bogotá, Colombia; ^2^ Department of Biomedical Engineering, Universidad de los Andes, Bogotá, Colombi; ^3^ Grupo CATALAD, Instituto de Química, Universidad de Antioquia, Medellín, Colombia; ^4^ Grupo de Diseño de Productos y Procesos (GDPP), Department of Chemical and Food Engineering, Universidad de los Andes, Bogotá, Colombia

**Keywords:** nanobioconjugate, Buforin II, OmpA, silica nanoparticles, fullerenol, cellular uptake, endosomal escape

## Abstract

The limited delivery of cargoes at the cellular level is a significant challenge for therapeutic strategies due to the presence of numerous biological barriers. By immobilizing the Buforin II (BUF-II) peptide and the OmpA protein on magnetite nanoparticles, a new family of cell-penetrating nanobioconjugates was developed in a previous study. We propose in this study to extend this strategy to silica nanoparticles (SNPs) and silanized fullerenol (F) as nanostructured supports for conjugating these potent cell-penetrating agents. The same molecule conjugated to distinct nanomaterials may interact with subcellular compartments differently. On the obtained nanobioconjugates (OmpA-SNPs, BUF-II-PEG_12_-SNPs, OmpA-F, and BUF-II-PEG_12_-F), physicochemical characterization was performed to evaluate their properties and confirm the conjugation of these translocating agents on the nanomaterials. The biocompatibility, toxicity, and internalization capacity of nanobioconjugates in Vero cells and THP-1 cells were evaluated *in vitro*. Nanobioconjugates had a high internalization capacity in these cells without affecting their viability, according to the findings. In addition, the nanobioconjugates exhibited negligible hemolytic activity and a low tendency to induce platelet aggregation. In addition, the nanobioconjugates exhibited distinct intracellular trafficking and endosomal escape behavior in these cell lines, indicating their potential for addressing the challenges of cytoplasmic drug delivery and the development of therapeutics for the treatment of lysosomal storage diseases. This study presents an innovative strategy for conjugating cell-penetrating agents using silica nanoparticles and silanized fullerenol as nanostructured supports, which has the potential to enhance the efficacy of cellular drug delivery.

## 1 Introduction

One of the significant obstacles to the safe and efficient delivery of pharmacological agents to the desired tissues or cells is the development of carriers that can pass through different biological barriers, such as the cellular membrane, while avoiding the immune response, side-target effects, or degradative pathways, to ultimately reach the target site while maintaining high availability of the therapeutic cargo (McNeil, 2011; McNeil, 2018).

Carriers based on nanoparticles (NPs) have been evaluated due to their multifunctionality, which results from their easily modifiable particle shape and size, material composition, and structure, according to the requirements of both the different cargoes and the target sites, achieving not only high biocompatibility, bioavailability, and biodistribution, but also on-target effects (Hossen et al., 2018; Karabasz et al., 2020). For instance, Planque et al. (2011) reported that membrane permeability and integrity are highly dependent on the size and surface chemistry of the NPs. Silica nanoparticles (SNPs) are one of the preferred nanomaterials for drug delivery due to their many advantageous properties. This material is an excellent candidate for drug carriers due to its high thermal stability, chemical inertness, high hydrophilicity and biocompatibility, simple functionalization and high loading capacity, and inexpensive synthesis (Gonçalves, 2018; Esim et al., 2019). Recently, SNPs have been utilized for the diagnostic and therapeutic delivery of contrast agents and drugs, biosensors, DNA carriers, and enzyme immobilization (Kim et al., 2019). Fullerenes, on the other hand, are an emerging class of carbon-based nanomaterials for cellular-level cargo delivery (Bolskar, 2013). These materials exhibit a structure with unique physicochemical properties and a highly symmetric cage with different sizes. The C_60_ fullerene has the most symmetrical structure. Fullerene-based systems have been used to investigate the release of chemotherapeutic agents to eliminate the side effects of drugs such as doxorubicin and paclitaxel, photosensitizers for the activation of reactive oxygen species for the treatment of cancer cells, nucleic acid release, drugs with anti-HIV-1 activity, transdermal release, fullerenols with antioxidant activity, cardiovascular drugs and release in the brain (Kazemzadeh and Mozafari, 2019).

In addition to the use of nanomaterials, known cell penetration agents, such as the protein OmpA (López-Barbosa et al., 2019) or the antimicrobial peptide BUF-II (Cuellar et al., 2018), are also used to increase the membrane permeability of drugs. These agents have the ability to translocate across biological barriers such as the cell membrane or even the blood–brain barrier (Komin et al., 2017). However, these molecules lack stability and have a short lifetime in biological systems, a problem that can be resolved by immobilizing them on nanomaterials (Alves and Olívia Pereira, 2014). Over the past few years, we have developed a dual strategy to engineer the surface of nanocarriers. This strategy involves functionalizing them with cell-penetrating agents and combining their attributes to create carriers that are more stable and have a higher loading capacity for therapeutic agents. By doing so, we aim to enhance the release of therapeutic agents from these carriers. The purpose of this study is to examine the effect of changing the nanostructured support on the translocation capacity and endosomal escape ability of cell-penetrating agents. To accomplish this, we intend to combine our knowledge of SNPs and fullerenol as potential nanostructured supports for conjugating these agents. Our goal is to determine if the resulting nanobioconjugates have the potential for efficient cell penetration and endosomal escape, which is essential for the success of many drug therapies.

Overall, the objective of our research is to determine the efficacy of various nanostructured supports in enhancing the performance of cell-penetrating agents. By investigating the translocation and endosomal escape ability of these agents, we hope to gain insights that will lead to the future development of more effective drug therapies.

## 2 Materials and methods

### 2.1 Materials

Tetraethylorthosilicate (TEOS) (98%), methanol, ammonia solution (30%–32%), tetramethylammonium hydroxide (TMAH) (25%), (3-Aminopropyl)triethoxysilane (APTES) (98%), glutaraldehyde (25%), amine-PEG_12_-propionic acid, N-hydroxysuccinimide (NHS) (98%), N-[3-dimethylammino)-propyl]-N′-ethyl carbodiimide hydrochloride (EDC) (98%), dimethyl sulfoxide (DMSO), Fullerene C_60_, Tetra-n-butylammonium hydroxide (TBAH) (40% in water), toluene, hydrogen peroxide (H_2_O_2_), glacial acetic acid, 2-propanol, diethyl ether, and hexane were purchased from Sigma-Aldrich (MO, United States). Buforin II (BUF-II-TRSSRAGLQFPVGRVHRLLRK) was purchased from GL Biochem Shanghai (Shanghai, China). Vero Cells (ATCC^®^ CCL-81) and THP-1 Cells (ATCC^®^ TIB-202) were used for delivery assays. MTT (3-[4,5-Dimethylthiazol-2-yl]-2,5-Diphenyltetrazolium Bromide), DAPI (4′,6-diamidino-2-phenylindole, dihydrochloride), and Lysotracker Green DND-26 was purchased from Thermo Scientific (MA, United States). Dulbecco’s modification of Eagle medium (DMEM), Roswell Park Memorial Institute (RPMI) 1640 medium, and fetal bovine serum (FBS) were obtained from Biowest (MO, United States).

### 2.2 OmpA overexpression in *E. coli*

OmpA protein was obtained from overexpression in *Escherichia coli*, following the protocol developed by Aguilera-Segura et al. (2014). *E. coli* K-12 W3110/pCA24N OmpA+34 was grown in Luria-Bertani (LB) agar plates [yeast extract (5 g L^−1^), bacto tryptone (10 g L^−1^), NaCl (10 g L^−1^)] supplemented with chloramphenicol (50 μg mL^−1^), and incubated for 16 h at 37°C, 250 rpm. Fresh liquid LB medium (19.5 mL) was inoculated with 500 μL from the previous culture and incubated at 37°C, 250 rpm, until reaching an optical density of 0.7 at 600 nm (OD600 nm). OmpA was obtained by inducing with IPTG (isopropylthio-β-galactoside) (2 mM) and by culturing for 3 more hours.

### 2.3 OmpA purification and characterization

The culture was centrifugated to obtain a pellet of OmpA overexpressed *E. coli*. The pellet was resuspended in buffer lysis in a ratio of 4 mL g^-1^, sonicated at 4°C for 40 min and 37% amplitude, and centrifuged at 13,000 rpm and 4°C for 15 min. Since OmpA protein was cloned with a His-tag, purification was attained by exposing the recovered supernatant to the Dynabeads^®^ TALON^®^ kit (Invitrogen). Purified OmpA protein was verified by SDS-PAGE, which showed a single 31 kDa band that agrees well with the molecular weight of OmpA. Concentration was measured via NanoDrop Spectrophotometer (Thermo Fisher Scientific) at 280 nm.

### 2.4 Synthesis and silanization of SNPs

**FIGURE 1:**
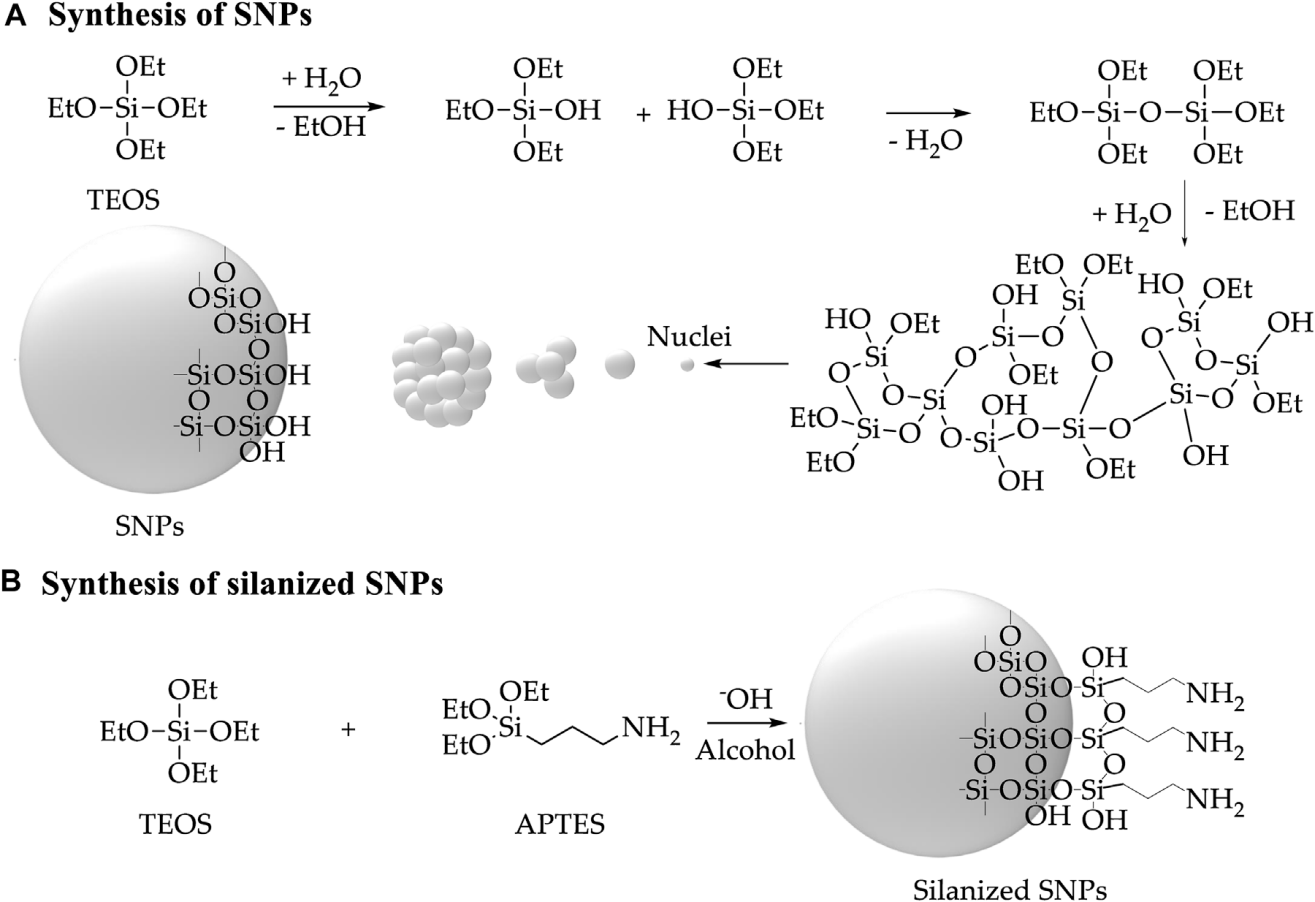
Schematic of the synthesis and silanization of SNPs **(A)** Synthesis of SNPs by TEOS hydrolysis in alcohol, water, and ammonia solution **(B)** Silanization of SNPs by TEOS hydrolysis in alcohol, water, and ammonia solution with the addition of APTES (Masalov et al., 2011; Shafqat et al., 2019).

SNPs were synthesized based on a Stober-like approach. The method involves hydrolysis and polycondensation of TEOS in an alcohol, water, and ammonia solution (Figure 1A) (Stober et al., 1968; Edrissi et al., 2011). Briefly, Ultrapure (type I) water (ultrapure water with a resistivity> 18 MΩ-cm and conductivity <0.056 μS cm^−1^) (1.5 mL) and methanol (66.3 mL) were mixed. TEOS (0.9 mL) was then added and sonicated for 20 min using an ultrasonic bath (Elmasonic EASY 60H, 37 kHz, 150 W), then 30% ammonia in an aqueous solution (4.5 mL) was added, and the mixture was left in ultrasound for another 60 min in which a cloudy white suspension formed. The SNPs were centrifuged (Z-216, Hermle Labortechnik GmbH, German) and washed with Ultrapure (type I) water (3 × 20 min, 14,500 rpm). Silanized SNPs were synthesized using a ratio of TEOS 95% and APTES 5% (Figure 1B) (Shafqat et al., 2019). Silanization with APTES renders aminopropyl functionalities on the surface of the NPs, which can be used to conjugate further BUF-II and OmpA or crosslinkers to generate reactive groups for coupling them. The silanized SNPs were centrifuged (Z-216, Hermle Labortechnik GmbH, German) (4 × 20 min, 14,500 rpm) and washed with Ultrapure (type I) water. BUF-II and OmpA were conjugated according to the calculations presented in Supplementary Data S1 (Rangel-Muñoz et al., 2020).

### 2.5 Synthesis and silanization of fullerenol

Fullerenol was prepared from fullerene C_60_ by hydroxylation with H_2_O_2_ and TBAH as a phase transfer catalyst under organic-aqueous bilayer conditions (Kokubo et al., 2011). Briefly, to a solution of fullerene C_60_ (100 mg) in toluene (50 mL), an aqueous solution of 30% H_2_O_2_ (10 mL) and TBAH (40% in water, 500 µL) was added and stirred for 16 h at 60°C. Subsequently, to eliminate residual TBAH, the aqueous phase containing the fullerenol was separated, and fullerenol was precipitated with a mix of 2-propanol, diethyl ether, and hexane (7:5:5, 85 mL). Then, to complete the purification, we combined dialysis (cellulose membrane dialysis tubing) and freeze-drying (Conversion: 100%, yield after purification: 75%) (De Santiago et al., 2019). Next, fullerenol (50 mg) was dissolved in 15 mL of Ultrapure (type I) water. TMAH solution (500 μl, 25% (v/v)) and glacial acetic acid (25 μL) were then added to the solution and sonicated for 10 min. APTES solution (500 μl, 20% (v/v)) was added to the fullerenol solution for silanization. The silanized fullerenol was washed with Ultrapure (type I) water to remove the APTES that was not covalently attached to the fullerenol.

### 2.6 BUF-II and OmpA bioconjugation

**FIGURE 2:**
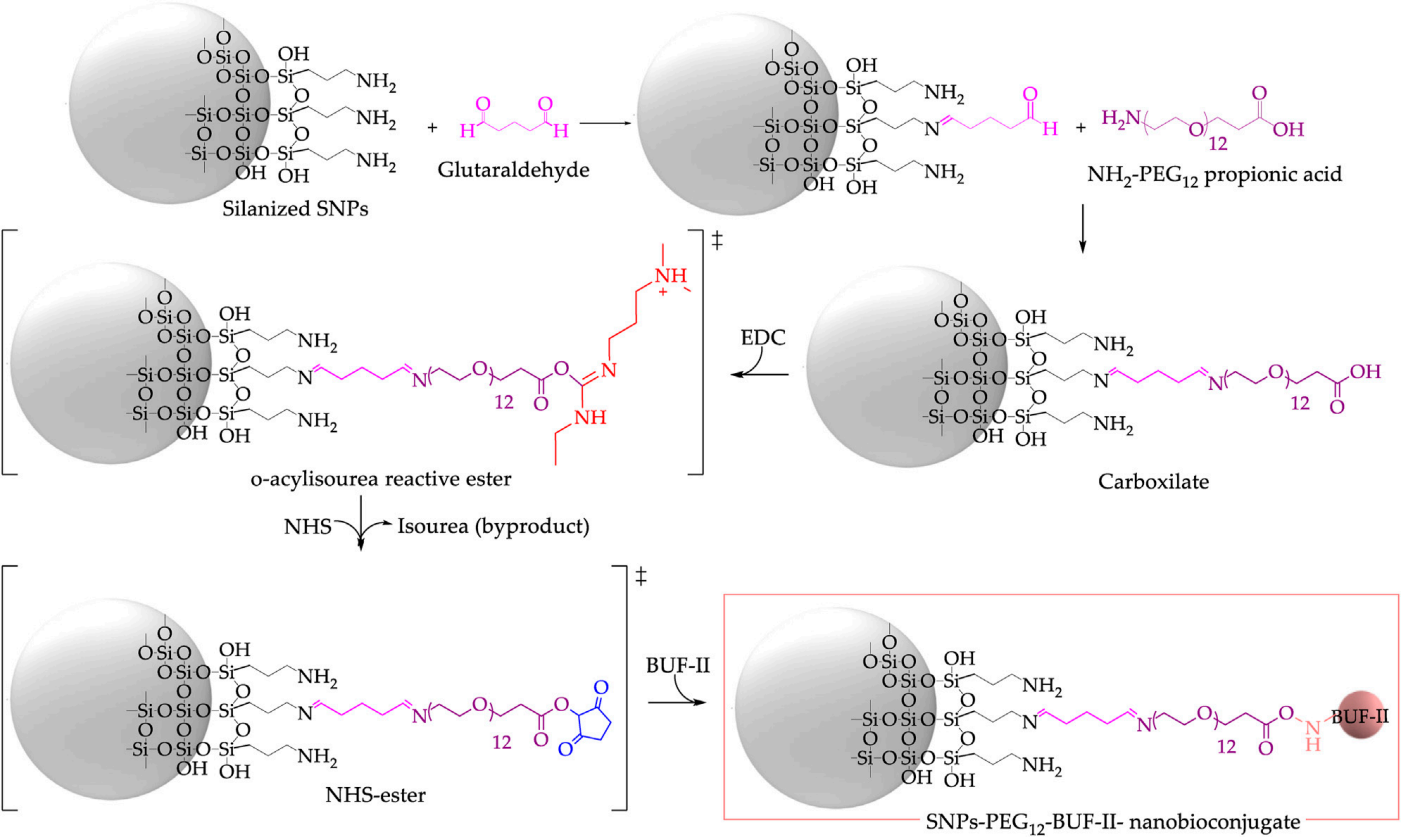
Synthesis of SNPs-PEG_12_-BUF-II nanobioconjugates via a multi-step reaction involving glutaraldehyde, amine-PEG_12_-propionic acid, and EDC/NHS to form an amide bond between a carboxylate and N-terminal of the peptide BUF-II.

Briefly, 100 mg of silanized SNPs or fullerenol were suspended in 30 mL of Ultrapure (type I) water and sonicated for 10 min (Elmasonic EASY 60H, 37 kHz, 150 W). This was followed by adding 2 mL of glutaraldehyde 2% (v/v) and by letting the mixture left to react in an orbital shaker for 1 h at 220 rpm. The amine-PEG_12_-propionic acid spacer was utilized to impart flexibility to BUF-II conjugated to SNPs or fullerenol, thereby increasing the probability of interaction with the target sites. After adding 10 mg of amine-PEG_12_-propionic acid, the mixture was shaken for 24 h at 220 rpm. Finally, 100 mg of functionalized nanomaterial was resuspended in 30 mL of type I Ultrapure water. BUF-II was conjugated to the carboxyl groups of the spacer by its N-terminal using two equivalents of EDC and two equivalents of NHS (concerning the carboxyl groups) (Figure 2; Supplementary Figure S1). BUF-II (1 mg BUF-II per 100 mg of functionalized nanomaterial) was added and the mixture was shaken at 220 rpm for 24 h. The obtained nanobioconjugates were centrifuged (Z-216, Hermle Labortechnik GmbH, German) (4 × 20 min, 14,500 rpm) and washed with Ultrapure (type I) water (Cuellar et al., 2018; Perez et al., 2019; Ramírez-Acosta et al., 2020).

**FIGURE 3:**
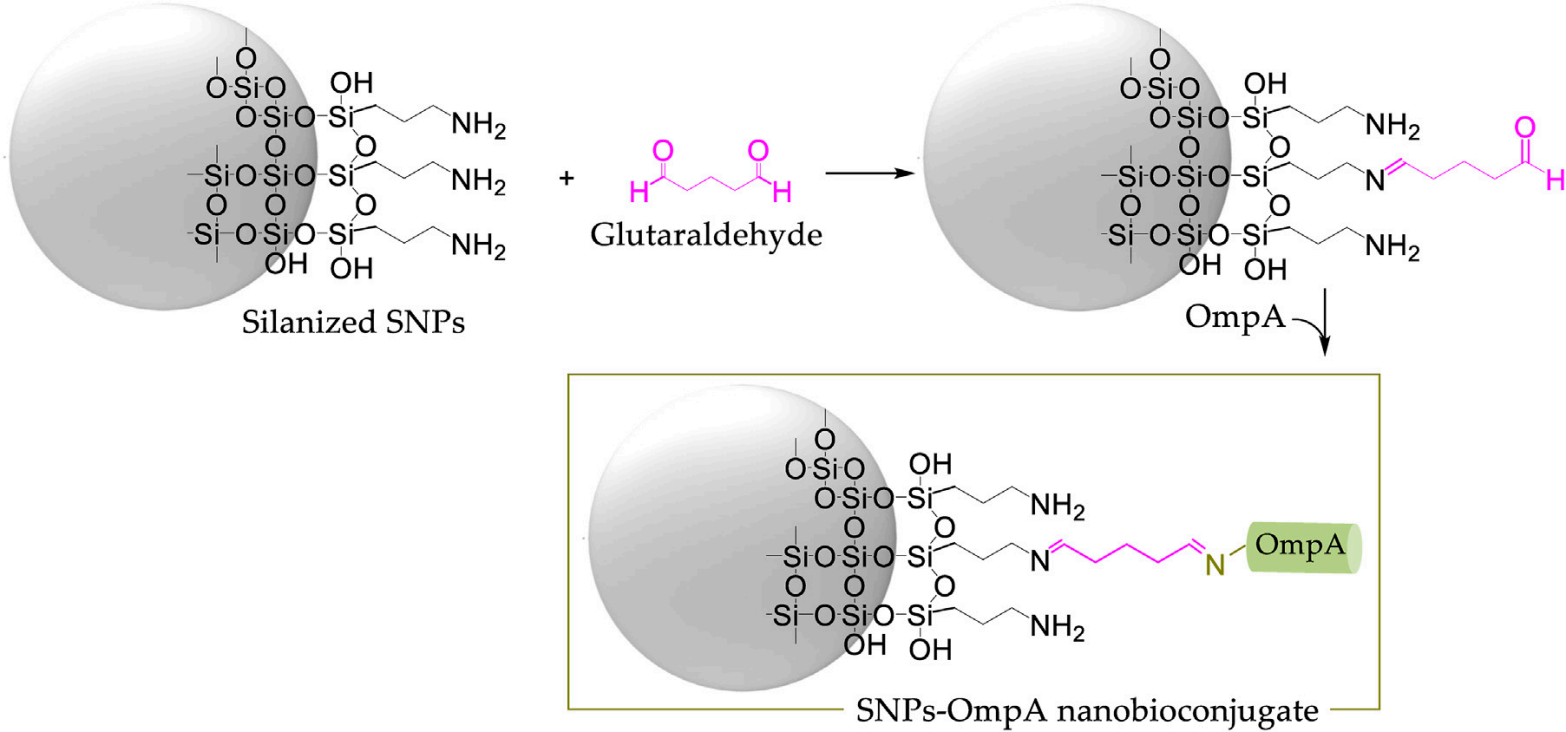
Synthesis of SNPs-OmpA nanobioconjugates using glutaraldehyde as the crosslinking agent.

Crosslinking of amine-terminal groups in the protein with aminopropyl groups on the surface of silanized SNPs or fullerenol facilitated by the addition of glutaraldehyde as the crosslinking agent enabled immobilization of OmpA on SNPs or fullerenol (Figure 3; Supplementary Figure S2) (López-Barbosa et al., 2019; Rangel-Muñoz et al., 2020). Briefly, 100 mg of silanized SNPs or fullerenol were suspended in 30 mL of ultrapure (type I) water and sonicated for 10 min (Elmasonic EASY 60H, 37 kHz, 150 W). This was followed by adding 2 mL of glutaraldehyde 2% (v/v) and by letting the mixture left to react in an orbital shaker for 1 h at 220 rpm. Then, OmpA (30 mg OmpA/100 mg functionalized nanomaterial) was added and shaken for 24 h at 220 rpm. The obtained nanobioconjugates were centrifuged (Z-216, Hermle Labortechnik GmbH, German) (4 × 20 min, 14,500 rpm) and washed with ultrapure (type I) water.

### 2.7 Labeling of nanobioconjugates with rhodamine B

For confocal microscopy evaluation of cellular uptake and endosomal escape, the nanobioconjugates were labeled with the fluorescent probe rhodamine B. This was accomplished through the formation of amide bonds between the carboxylate group of rhodamine B and the free amine groups of nanobioconjugates. Briefly, under dark conditions, 15 mg of EDC, 7.5 mg of NHS, and 1 mL of DMF were added to 5 mL of type I ultrapure water. Subsequently, 2 mg of rhodamine B was added, and the solution was heated to 40°C for 15 min with continuous magnetic stirring. This enables the activation of the carboxylate groups of rhodamine B to form amide bonds with the free amine groups of nanobioconjugates. The mixture was then allowed to cool to room temperature before being combined with 50 mg of nanobioconjugates. To prevent photobleaching, it was stirred for 24 h at 220 rpm using a shaker at room temperature and in complete darkness. The labeled nanobioconjugates were centrifuged (Z-216, Hermle Labortechnik GmbH, German) (20 min, 14,500 rpm) and washed several times with ultrapure (type I) water until no rhodamine B was detected in the supernatant (López-Barbosa et al., 2019).

### 2.8 Characterization of the nanobioconjugates

Infrared spectra were collected from 4,000–500 cm^−1^ with a spectral resolution of 2 cm^−1^ using a spectrometer ALPHA II FTIR Eco-ATR (Bruker Optik GmbH, Ettlingen, Germany) and an IRAffinity-1 spectrometer (Shimadzu Corporation). The hydrodynamic diameter and *ζ* potential of the nanobioconjugates were determined via Dynamic Light Scattering (DLS) and Electrophoretic Moobility (Zeta-Sizer Nano-ZS; Malvern Instruments, Malvern, UK). Thermogravimetric analysis (TGA, TA Instruments, New Castle, DE, United States) was used to estimate the amount of material conjugated to the SNPs and the fullerenol, implementing a linear temperature ramp at a rate of 10°C min^−1^ from 25°C to 890°C under an inert atmosphere. Focused Ion Beam Scanning Electron Microscope (TESCAN LYRA3 FIB-SEM, Czech Republic) and Transmission Electron Microscope (TEM, FEI TECNAI G2 F20 Super Twin TMP, Hillsboro, OR, United States) were used to obtain information on the size, composition, and morphology of the nanomaterials. XPS spectra were obtained using a SPECS near-ambient pressure X-ray photoelectron spectrometer (NAP-XPS) with a PHOIBOS 150 1D-DLD analyzer, using a monochromatic source of Al-Kα (1,486.7 eV, 13 kV, 100 W) (SPECS GmbH, Berlin, Germany). The X-ray source and monochromator were aligned to get a 0.49 eV peak-resolution under a vacuum pressure of the chamber below 10^−9^ m bar. The samples were previously mounted on a non-conductive tape. The control of surface potential was achieved by an electron flood gun at 3 kV over a tantalum mesh with a nominal aperture of 0.43 mm. The spot size was 200 nm of diameter, the energy pass was fixed at 20 eV and the scan number for the high-resolution measurements was 20. The signals were calibrated to a binding energy of 284.6 eV for adventitious carbon and a Ta4f7/2 peak from the tantalum mesh was employed as reference. XPSPeak software was used for fitting the XPS spectra using a Shirley-type single-peaks background with a simultaneous GL peak-shape of 30% and full-width at half maximum (FWHM) data from literature.

Delivery of nanobioconjugates in mammalian cells was monitored using a confocal laser scanning microscope (Fluoview FV1000, Olympus, Tokyo, Japan). The images were obtained with a UPLSAPO 20x/0.75 NA objective and a PlanApo ×60/1.35 NA objective. Excitation/Emission wavelengths of 405/422, 488/520, and 559 nm/575 nm were used to detect DAPI (nuclei), LysoTracker green (acidic organelles: endosomes/lysosomes), and rhodamine B (nanobioconjugates), respectively. Colocalization within biologically relevant regions of interest (ROIs) was analyzed using the plugin Coloc 2 of the Fiji^®^ software (https://imagej.net/Fiji/Downloads). At least 30 images were taken for each treatment (about 10 cells per image were analyzed).

### 2.9 *In vitro* analysis of the nanobioconjugates’ hemolytic effect

Hemolysis is the rapid destruction of erythrocyte membranes, which results in the release of intra-erythrocyte contents into the blood plasma. The hemolytic activity of the nanobioconjugates was determined using the method described by previously Muñoz-Camargo et al. (2018). Briefly, blood from healthy donors was collected in BD Vacutainer^®^ blood tubes using EDTA as an anticoagulant. The samples were obtained with the approval of the Ethical Committee at the Universidad de los Andes (minute number 928-2018). Blood was centrifuged at 1,800 rpm for 5 min (Micro Centrifuge Z 360, Hermle Laboratories GmbH) to collect red blood cells, and the hematocrit level (lower layer, red) and plasma (upper layer, yellow) were marked. The plasma was then removed, and the tube was refilled to the mark with 150 mM NaCl, inverting the tube gently to mix, and centrifuged again. Subsequently, the supernatant was removed and replaced with PBS (Phosphate Buffered Saline) (1X). A red blood cells stock was prepared by adding 1 mL of isolated red blood cells (4.3 × 10^6^ red blood cells μL^−1^) in 9 mL of PBS (1X). Serial dilutions of nanobioconjugates (300, 150, 75, 37.5, and 18.75 μg mL^−1^ in PBS) were prepared for the test in a 96-well microplate. 100 μL of each treatment and 100 µL of the diluted red blood cells were incubated for 1 h at 37°C and 5% CO_2_. Finally, the microplate was centrifuged at 1,800 rpm for 5 min, and 100 µL of each supernatant was measured (oxyhemoglobin, 450 nm) in a microplate reader spectrophotometer (Multiskan™ FC, Thermo Fisher Scientific Inc., United States). PBS (1X) and Triton X-100 (1%) were used as negative and positive controls, respectively.

### 2.10 *In vitro* assessment of nanobioconjugates’ blood coagulation effect

The effect of the nanobioconjugates on platelet aggregation was tested using platelet-rich plasma (PRP). Blood was obtained from healthy donors in BD Vacutainer^®^ tubes, anticoagulated with sodium citrate. PRP was obtained by centrifugation of a human blood sample at 1,000 rpm for 15 min (Micro Centrifuge Z 360, Hermle Labortechnik GmbH), and the PRP was collected and transferred to a fresh tube. Serial dilutions of nanobioconjugates (300, 150, 75, 37.5, and 18.75 μg mL^−1^ in PBS) were prepared for the test in a 96-well microplate. Subsequently, 100 µL of each treatment and 100 µL of PRP were incubated for 15 min at 37°C and 5% CO_2_. Thrombin (6U) was used as a positive control, while PBS (1X) was a negative reference. Finally, the aggregation was measured by optical density (OD) at 620 nm in a microplate reader spectrophotometer (Multiskan™ FC, Thermo Fisher Scientific Inc., United States) (Lopez-Barbosa et al., 2020).

### 2.11 MTT cytotoxicity test

The MTT (3-[4,5-dimethylthiazol-2-yl]-2.5 diphenyl tetrazolium bromide) assay is based on the metabolic reduction of MTT into formazan crystals by viable cells (Meerloo et al., 2011). Briefly, Vero cells (ATCC^®^ CCL-81) and THP1 cells (ATCC^®^ TIB-202) were plated in 96-well culture plates in DMEM (1.0 × 10^6^ cells 100 μL^−1^ per well) and incubated at 37°C and 5% CO_2_ for 24 h. Culture media was removed from wells, and DMEM 1% penicillin/streptomycin (90 μL) (without FBS) was added to each well. Subsequently, 100 µL of each treatment (300, 150, 75, 37.5, and 18.75 μg mL^−1^ in PBS) were added and incubated at 37°C, 5% CO_2_ for 24 h, and 48 h. The medium was removed, and DMSO (500 μL) was used to dilute formazan crystals. Absorbance was read at 595 nm (reference 650 nm) in a microplate reader spectrophotometer (Multiskan™ FC, Thermo Fisher Scientific Inc., United States) and compared to the controls (Lopez-Barbosa et al., 2020).

### 2.12 Cell translocation and endosome escape

Vero Cells were seeded in a sterile glass slide previously placed in a 6-well microplate and incubated in DMEM medium supplemented with 10% (v/v) FBS at 37°C and 5% CO_2_ for 24 h. Next, cells were exposed to fluorescently labeled nanobioconjugates (18.75 μg mL^-1^), and the samples were incubated for 30 min and 4 h at 37°C and 5% CO_2_. Supplemented culture medium was removed, and then, the cells were washed three times with DMEM medium and exposed for 10 min to DAPI (1 µL, 1:1,000) used to stain nuclei, and Lysotracker Green DND-26 (0.1 µL, 1:10,000) that labels acidic organelles (lysosomes/endosomes) before capturing confocal images (López-Barbosa et al., 2019). THP-1 Cells exposed to fluorescently labeled nanoconjugates (18.75 μg mL^-1^) were incubated for 30 min and 4 h at 37°C and 5% CO_2_. Then, the samples were exposed for 10 min to DAPI (1 µL, 1:1,000) and Lysotracker Green DND-26 (0.1 µL, 1:10,000) before capturing confocal images (López-Barbosa et al., 2019).

### 2.13 Statistical analysis

Values (Hemolysis, platelet aggregation, cell viability) are expressed as the means ± SDs of triplicates. Significance tests were analyzed by nonparametric—the normality of data distributions was assessed using the Shapiro–Wilk test—one-way ANOVA (Kruskal–Wallis test) and Dunn’s multiple comparison test, using the GraphPad Prism 8.0.1^®^ software (GraphPad Software, La Jolla, CA, United States). *p* values <0.05 were considered statistically significant.

## 3 Results and discussion

### 3.1 Physicochemical characterization of SNPs and nanobioconjugates based on SNPs

**FIGURE 4:**
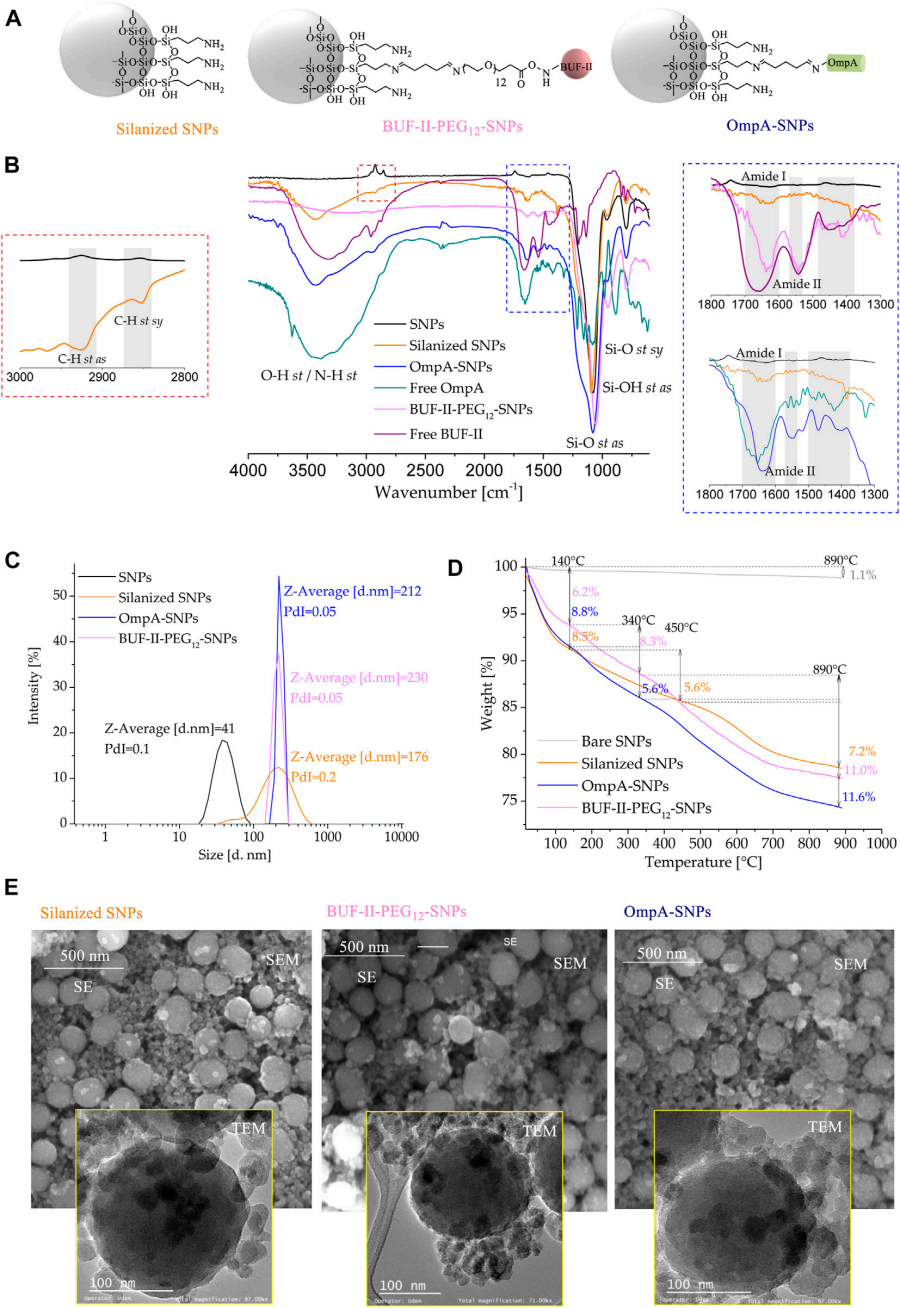
Spectroscopic and thermal analyses of silanized SNPs and the nanobioconjugates **(A)** Schematic of the chemical structure of silanized SNPs and the nanobioconjugates **(B)** FT-IR spectra of **(1)** bare SNPs, **(2)** silanized SNPs, **(3)** BUF-II-PEG_12_-SNPs nanobioconjugates, **(4)** free BUF-II, **(5)** OmpA-SNPs, and **(6)** free OmpA **(C)** DLS histogram for the size intensity distribution **(D)** TGA thermogram of SNPs, silanized SNPs, and nanobioconjugates **(E)** SEM and TEM images of the silanized SNPs, and nanobioconjugates.

Figure 4A shows a schematic of the chemical structure of silanized SNPs and BUF-II-PEG_12_-SNPs, and OmpA-SNPs nanobioconjugates. Figure 4B compares the FT-IR spectra of bare SNPs, silanized SNPs, free OmpA, free BUF-II, and nanobioconjugates. The bare SNPs exhibit distinctive absorptions at around 1,100 cm^−1^ (Si-O *st as*, asymmetrical stretching), 970 cm^−1^ (Si-OH *st as*), and 801 cm^−1^ (Si-O *st sy*, symmetrical) (Pretsch et al., 2009; Edrissi et al., 2011). New bands were observed at 2,925 cm^−1^ (C-H *st as*), 2,852 cm^−1^ (C-H *st sy*), and 1,639 cm^−1^ (N-H *b*, bending vibration), evidencing the presence of propylamine groups on the surface of the silanized SNPs (Shafqat et al., 2019). On the free OmpA, OmpA-SNPs, free BUF-II, and BUF-II-PEG_12_-SNPs spectra, the presence of amide vibrational modes known as amide I (1,700–1,600 cm^−1^) and amide II (1,570–1,540 cm^−1^) and other vibrations specific that are absent on bare SNPs suggests correct conjugation of protein or peptide (Pretsch et al., 2009; Tatulian, 2013; López-Barbosa et al., 2019). The amide I mode is generated mostly by contributions of the C=O *st*, CN *st*, CCN *ob* (out-of-plane bending), and by the NH *ib* (in-plane bending) modes (Tatulian, 2013). The amide II mode includes contributions of the NH *ib*, CN *st*, CO *ib*, CC *st*, and NC *st* (Tatulian, 2013). Other vibrational modes of the amide group of protein or peptide and crosslinking agents overlap with the vibrational modes of SNPs. Figure 4C shows particle size distribution by intensity determined by DLS. Bare SNPs exhibited a mean hydrodynamic diameter (Z-average size) of 41 nm with a Polydispersity Index (PdI) of 0.1. A ratio of 95% of TEOS and 5% of APTES produced two populations of silanized SNPs with mean hydrodynamic diameters around 40 and 220 nm (Z-average size: 176 nm, PdI: 0.2). Regarding this, Li et al. (2019) demonstrated that the total uptake of SNPs in Hela cells was higher in co-exposures of large and small SNPs — 50/100 and 80 nm/150 nm—than in single exposures of the same. It can also be observed that the Z-average size of the SNPs increased after peptide and protein conjugation for BUF-II-PEG_12_-SNPs (Z-average size: 212 nm, PdI: 0.05) and OmpA-SNPs (Z-average size: 230 nm, PdI: 0.05). There is no consensus regarding the optimal size that maximizes cellular uptake and maintains cell viability. A number of experimental studies indicate that particle size reduction does not necessarily increase cellular uptake (Barisik et al., 2014). However, nanocarriers based on NPs in the size range of 10–200 nm are frequently used to facilitate the delivery of cargoes at the cellular level. These nanocarriers are not easily excreted by any of the physiological systems designed for this purpose and therefore reach target organs and tissues in sufficient concentrations (Selby et al., 2017; Chenthamara et al., 2019). Thermogravimetric analysis (TGA) was used to estimate the amount of material conjugated to the SNPs (Figure 4D). The bare SNPs exhibited excellent thermal stability, losing only about 1.1% of their weight when heated from room temperature to 890°C. In contrast, weight loss was observed for silanized SNPs and nanobioconjugates in three temperature ranges (silanized SNPs: room temperature to 140°C, 140°C–450°C, and >450°C; nanobioconjugates: room temperature to 140°C, 140°C–340°C, and >340°C). Silanized SNPs, BUF-II-PEG_12_-SNPs, and OmpA-SNPs showed a first weight loss of 8.5%, 8.8%, and 6.2%, respectively, that can be attributed to water loss. A second weight loss of 5.6% was observed for the silanized SNPs and OmpA-SNPs, whereas for BUF-II-PEG_12_-SNPs, it was 8.3%. These losses can be assigned to the presence of non-hydrolyzed ethoxy groups of APTES and residual alcohol within the silica nanostructure (Kunc et al., 2019). Finally, the weight loss at the highest temperatures can be assigned to the loss of aminopropyl groups (7.2%) for the silanized SNPs, and the detachment of BUF-II, OmpA, and crosslinking agents in the case of the BUF-II-PEG_12_-SNPs (11.0%) and OmpA-SNPs (11.6%) nanobioconjugates (López-Barbosa et al., 2019; Perez et al., 2019). SEM and TEM images were consistent with the data obtained by DLS regarding the size and the presence of two size populations of particles. In addition, it can be observed that the nanoparticles have a predominantly spherical morphology. Apparently, after conjugation, the roughness of the nanoparticles changes; this could also affect their interaction with cells and their loading capacity (Niu et al., 2015; Alan et al., 2020) (Figure 4E). Additionally, *ζ* potential is indicative of the stability of the suspension; if all the particles in suspension have *ζ* potentials above +25 mV or below −25 mV, they repel each other, and therefore show no tendency for aggregation, coagulation, or flocculation (Shnoudeh et al., 2019). The *ζ* potential measurements of the SNPs indicate that in aqueous media—pH close to 7— it acquires a negative surface charge of −37.6 ± 4.91 mV; this value indicates good SNPs stability in water. The *ζ*-potential reached values of 4.41 ± 3.27 mV, 7.34 ± 3.36 mV, and 18.1 ± 5.13 mV for the silanized SNPs, and BUF-II-PEG_12_-SNPs, and OmpA-SNPs nanobioconjugates (in aqueous media—pH close to 7), respectively. At physiological pH, these nanobioconjugates tend to precipitate.

**FIGURE 5:**
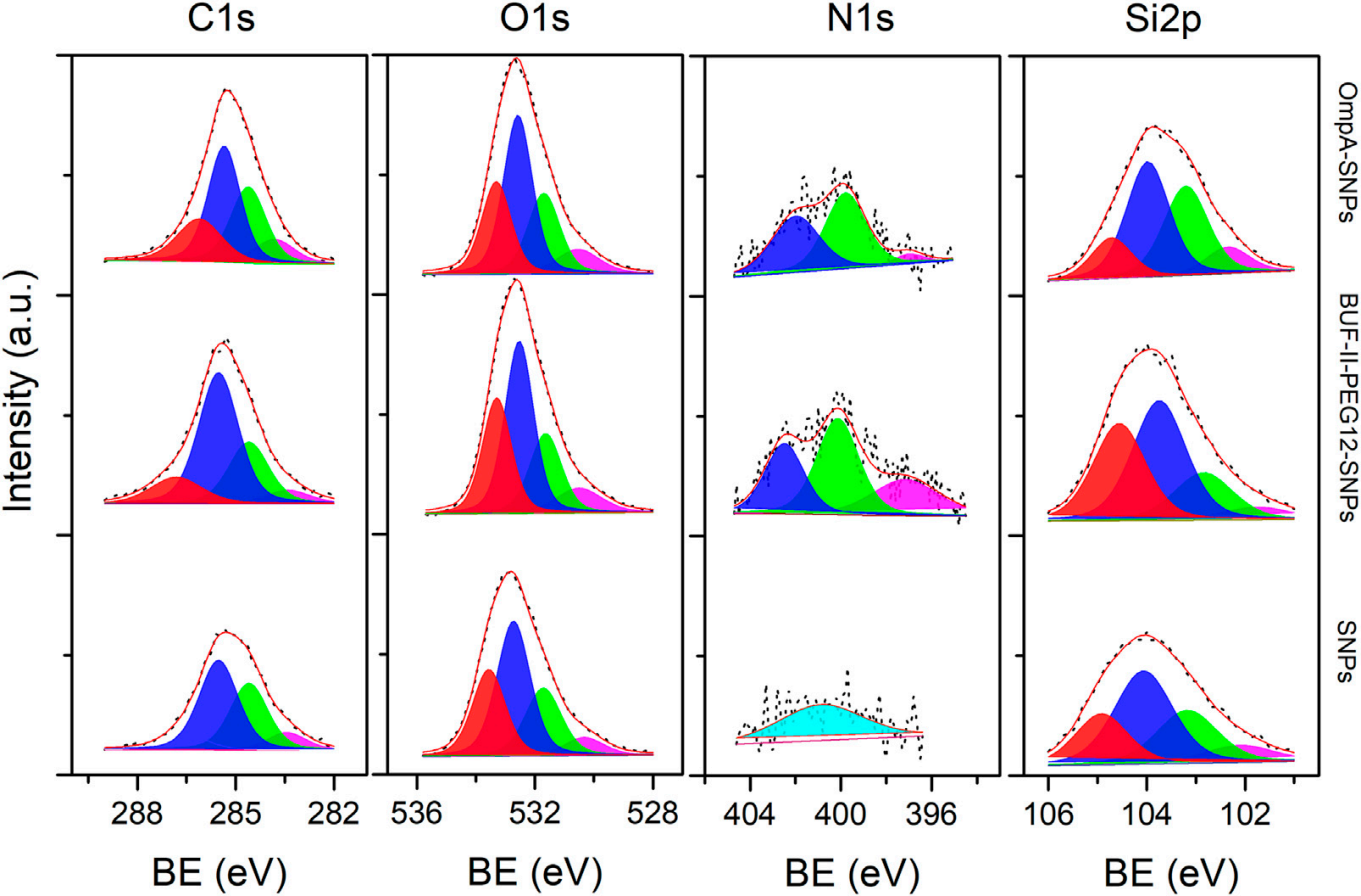
XPS spectra of the C1s, O1s, N1s, and Si2p (left to right) core-level regions of silanized SNPs, BUF-II-PEG12-SNPs nanobioconjugates, and OmpA-SNPs nanobioconjugates samples (bottom to top). Peak components for the XPS lines are differentiated by colors from high to low binding energy values (left to right).

The chemical surface characterization of the nanobioconjugates was evaluated by XPS. The detailed experimental set-up carried out for the samples is shown in Section 2.8. Here, SNPs and fullerenol nanocarriers were considered. Figure 5 shows the high-resolution (HR) spectra for the SNPs and the corresponding nanobioconjugates under examination. The peak components from the decomposition analysis are denoted from high to low binding energy, and colored zones clearly distinguish them. The binding energy (BE) values for all components that are part of the overall fitting, marked as a red line over black dots related to the experimental recorded data, are shown in Table 1. Starting at the C1s core-level, four mean sub-peaks for functionalizing samples were fitted, which correspond to O-C-O/C=O (red); C-O/C-N (blue); C-C (green) and C-Si (magenta) bonds. For the silanized SNPs, there were no highly oxidized species, which clearly indicate the successful conjugation of OmpA and BUF-II on the NPs’ surface. Since these biomolecules and their intermediate states are too complex due to their chemistry and molecular weight (MW), it is not possible to establish a stoichiometric ratio between the species; nevertheless, the counts (*Y*-axis) for each core-level were normalized prior to their placement on the plots. Conversely, a qualitative analysis can be done. As a result, it is possible to determine that the oxidizing species for C1s core-level in the OmpA-SNPs system are greater than those in the BUF-II-PEG12-SNPs nanobioconjugates. In contrast, the BUF-II-PEG12-SNPs exhibited a high (C-N/C-O)/C-C ratio due to their low molecular weight and high C-N/C-O terminal bonds. Consistent C-Si bonds were found in the studied systems, allowing us to conclude that the SNPs were properly silanized and that the biomolecules are bound to the inorganic nanoparticles via C-Si-O covalent bonds. The O1s core-level was deconvoluted into four mean sub-peak components associated with chemisorbed OH- molecules: O-C-O/Si-O, C-O, and O=C species. Slight shifts in binding energies and high similitudes of the integral intensity of every peak as calculated from the area under the curve were observed. Moreover, if we compare the overall peak intensity with that of C1s for each compound, the corresponding ratio for the nanobioconjugates is lower than for the SNPs. This is most likely due to the lower concentration of C/O species on the surface of SNPs, where only the APTES chain is present. As peptide and protein are conjugated, the proportion of atomic C species increases dramatically, as does the C/O ratio. Now passing through the N1s core-level, three mean sub-peak components for nanobioconjugates were fitted. In the case of silanized SNPs, a weak signal of nitrogen from the conjugated APTES can be assigned to a primary amine (Talavera-Pech et al., 2016). For the nanobioconjugates, protonated amines seem to be located at higher binding energies, followed by O- C-N and N=N-/N-H- bonds. The Si2p sub-peaks components corresponding to SiOx (red), Si-O- (blue), Si-O-C- (green) and Si-C- (magenta) bonds are shown from left to right for bare SNPs and nanobioconjugates (Talavera-Pech et al., 2016). Slight peak shifts and intensity changes can be attributed to conjugation of OmpA and BUF-II and are likely related to conformational changes upon conjugation.

**TABLE 1:** Binding energy (BE) of the different XPS lines for C1s, O1s, N1s, and S2p peak components from silanized SNPs, BUF-II-PEG12-SNPs nanobioconjugates, and OmpA-SNPs nanobioconjugates samples.

Sample	C1s (BE- eV)	O1s (BE- eV)	N1s (BE- eV)	Si2p (BE- eV)
Silanized SNPs	283.4	530.3	400.8	102.0
	284.6	531.7	—	103.2
	285.5	532.7	—	104.1
	286.5	533.6	—	104.9
BUF-II-PEG_12_-SNPs	283.3	530.5	397.2	101.8
	284.6	531.6	400.1	102.8
	285.5	532.5	402.5	103.7
	286.8	533.3	—	104.6
SNPs-OmpA	283.8	530.5	396.9	102.3
	284.6	531.7	399.8	103.2
	285.4	532.6	401.9	104.0
	286.1	533.,3	—	104.7

### 3.2 Physicochemical characterization of fullerenol and fullerenol-based nanobioconjugates

**FIGURE 6:**
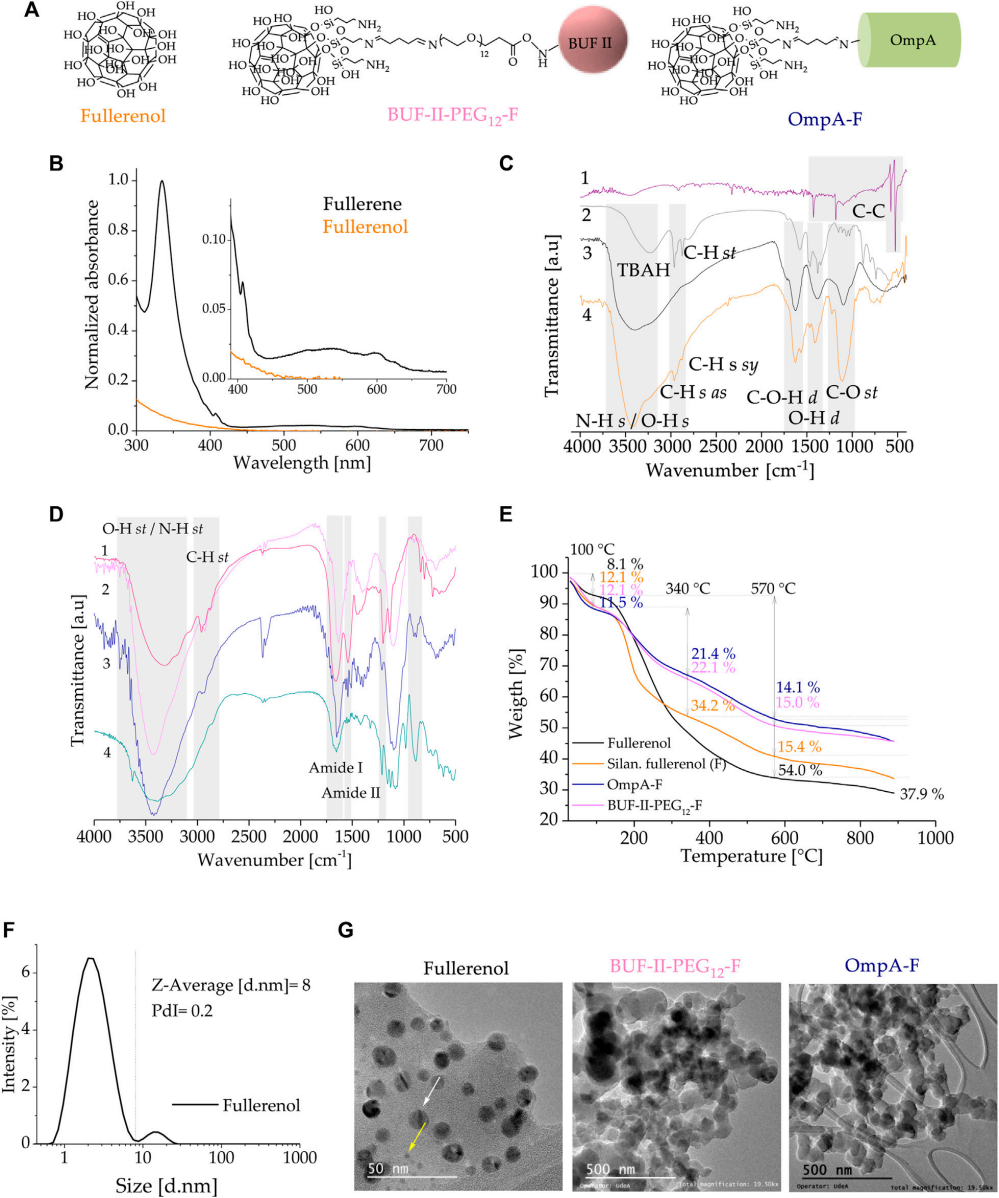
Spectroscopic and thermal analyses of fullerenol and the nanobioconjugates **(A)** UV–VIS spectra of C_60_ in toluene and aqueous solution of fullerenol **(B)** Schematic of the chemical structure of fullerenol and the nanobioconjugates **(C)** FT-IR spectra of (1) fullerene, (2) fullerenol as produced (with TBAH residues), (3) purified fullerenol, and (4) silanized fullerenol **(D)** FT-IR spectra of (1) BUF-II-PEG12-F nanobioconjugates, (2) free fullerenol, silanized fullerenol, and nanobioconjugates **(E)** TGA thermogram of fullerenol, silanized fullerenol, and nanobioconjugates **(F)** DLS histogram for the size intensity distribution **(G)** TEM images of the fullerenol, and nanobioconjugates.

Figure 6A shows a schematic of the chemical structure of silanized fullerenol, OmpA-F, and BUF-II-PEG_12_-F nanobioconjugates. Figure 6B shows UV–visible absorption spectra of fullerene C_60_ in toluene and fullerenol in water. C_60_ fullerene dissolved in toluene has characteristic absorption bands with maxima at 283, 335, and 408 nm, followed by a broad absorption band in the range of 430–650 nm with reduced absorptions for the blue region and red; this combination gives the compound its distinctive purple color (Ajie et al., 1990). Fullerenol dissolved in water is yellow and almost transparent in the visible region due to its considerable perturbation of the π-conjugation upon hydroxylation (Kokubo et al., 2008). Figure 6C compares the FT-IR spectra of (1) fullerene, (2) fullerenol as synthesized (with TBAH residues), (3) purified fullerenol, and (4) silanized fullerenol. Fullerene C_60_ has four active infrared modes at 1,429, 1,182, 573, and 525 cm^−1^ due to C-C bonds (Krätschmer et al., 1990). In the as-synthesized fullerenol, the two peaks observed at 2,963 and 2,873 cm^–1^ (C-H *st*) were attributed to residual TBAH (Kokubo et al., 2011). Purified fullerenol showed a broad band at around 3,424 cm^–1^(O-H *st*) and four characteristic bands at 1,598 cm^–1^ (C=C *st*), 1,410 cm^–1^ (O-H *b*), 1,352 cm^–1^ (C-O-H *b*), and 1,112 cm^–1^ (C-O *st*), which agree well with previously reported data (Kokubo et al., 2011; Ravelo-Nieto et al., 2020). Silanization was confirmed by the presence of new bands at 2,964 cm^–1^ (C-H *st as*), 2,934 cm^–1^ (C-H *st sy*), 2,875 cm^–1^ (H-C (-N) *st*), 1,564 cm^–1^ (N-H *b*), and 1,344 cm^–1^ (C-N *st*), which can be attributed to propylamine groups. Moreover, absorption bands at 1,653 cm^–1^ (N-H *b*), 1,110 cm^–1^ (Si-O *st*), 1,052 cm^–1^ (Si-O-Si *st*), and 690 cm^–1^ (Si-C *st*) overlap with the vibrational modes of fullerenol (Cuellar et al., 2018). Figure 6D compares the FT-IR spectra of (1) OmpA-F, (2) free OmpA, (3) BUF-II-PEG_12_-F, and (4) free BUF-II (Shafqat et al., 2019). The free OmpA, OmpA-F, free BUF-II, and BUF-II-PEG_12_-F spectra showed the amide I and amide II vibrational modes along with other specific vibrations that are absent in the non-functionalized fullerenol. However, these signals overlap with the vibrational modes of fullerenol. Thermal stability of fullerenol and nanobioconjugates was studied by TGA (Figure 6E). TGA results of purified fullerenol show three main a weight loss stages: room temperature to 100°C, 100°C–570°C, and >570°C. The initial weight loss (∼8.1%) corresponds to dehydration of the samples (Kokubo et al., 2011). The second weight loss (∼54.0%) corresponds to the dehydroxylation before the structural degradation of the fullerene nucleus that occurs at temperatures above 570°C (∼37.9% residual weight) (Goswami et al., 2004). Then, using the method described by Goswami et al.( 2004), the number of −OH groups per fullerene could be estimated at 30, which is similar to results reported by others previously (Kokubo et al., 2011; Kovač et al., 2018; De Santiago et al., 2019). Four main weight loss steps are observed in silanized fullerenol, and BUF-II-PEG_12_-F, OmpA-F nanobioconjugates: room temperature to 120°C, 120°C–340°C, 340°C–570°C and >570°C. Silanized fullerenol, BUF-II-PEG_12_-F, and OmpA-F presented a first weight loss of ∼12.1%, ∼11.5%, and ∼12.1%, respectively. These can be attributed to the dehydration of the samples. The second weight loss of ∼34% was observed for the silanized fullerenol, whereas for BUF-II-PEG_12_-F and OmpA-F were ∼21% and ∼22%, respectively. Silanized fullerenol, BUF-II-PEG_12_-F, and OmpA-F presented a third weight loss of ∼15.4%, ∼14.1%, and ∼15.0%, respectively. These losses in the temperature range of 150°C–570°C can be assigned to the decomposition of aminopropyl groups for the silanized fullerenol and the detachment of the aminopropyl groups, conjugating agents and the BUF-II and OmpA for the nanobioconjugates (Goswami et al., 2004; Cuellar et al., 2018; Perez et al., 2019). Figure 6F shows the particle size distribution by intensity determined by DLS. The fullerenols should have a diameter of ∼1.0 nm but tend to form clusters in water easily (Brant et al., 2007; Kokubo et al., 2011). The synthesized fullerenol, re-dispersed by sonication, exhibited two populations of clusters with mean hydrodynamic diameters at around 2 nm and 14 nm (Z-average size: 8 nm, PdI: 0.2). After peptide and protein conjugation, the polydispersity of the samples increased and rendered them unsuitable for DLS measurements; consequently, we performed TEM analysis. (Figure 6G). Fullerenol TEM images were consistent with DLS data regarding cluster size and the presence of two cluster population sizes. Furthermore, a change in the morphology of the nanobioconjugates is evidenced after peptide and protein immobilization, as well as aggregate formation. The aggregation may be attributable to the use of glutaraldehyde, a bifunctional reagent with a propensity for uncontrolled polymerization during the conjugation process (Hermanson, 2013). The *ζ* potential measurements of the fullerenol indicate that in aqueous media—pH close to 7— it acquires a negative surface charge of −20.4 ± 7.47 mV. The *ζ*-potential varied to −12.9 ± 0.40 mV and −19.9 ± 0.65 mV for BUF-II-PEG_12_-F and OmpA-F nanobioconjugates in aqueous media—pH close to 7 —, respectively. These nanobioconjugates tend to precipitate at physiological pH.

**FIGURE 7:**
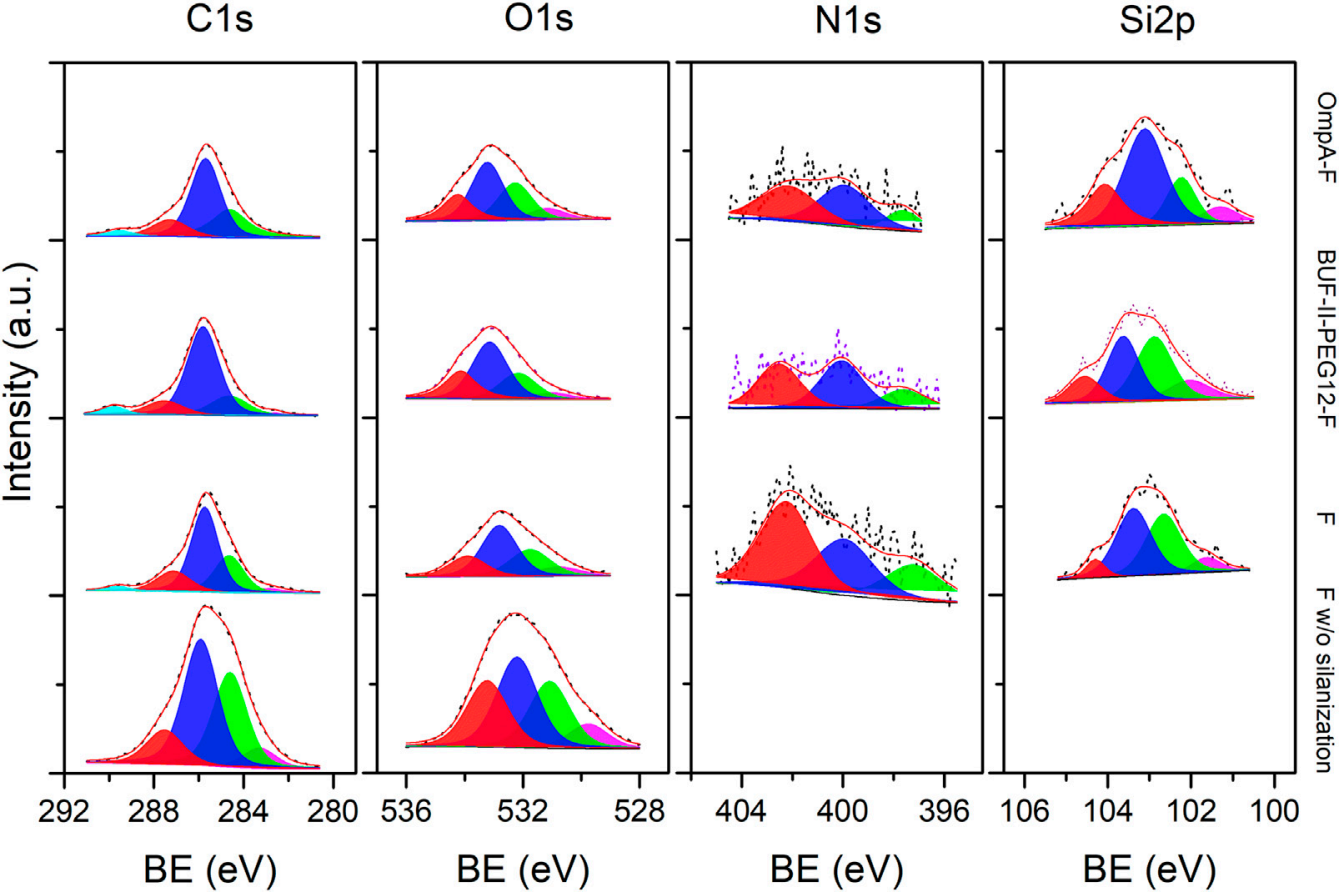
XPS spectra of the C1s, O1s, N1s and Si2p (left to right) core-level regions of Fullerenol, Silanized fullerenol (F), BUF-II-F and OmpA-F nanobioconjugates samples (bottom to top). Peak components for the XPS lines are differentiated by colors from high to low binding energy values (left to right).

Figure 7 shows the high-resolution (HR) spectra of fullerenol and the corresponding nanobioconjugates under examination. The decomposed peak components are labeled from high to low binding energy and depicted by colored zones. The color code employed is similar to that utilized for SNPs. Table 2 presents the binding energy (BE) values for all components integrated in the overall fitting, represented as a red line over black dots related to the experimental recorded data. Starting with the C1s core-level, the deconvolution revealed a sub-peak at higher energies and the presence of the C-C cage at 284.6 eV (Nurzynska et al., 2022). Following silanization and conjugation of BUF-II or OmpA, the peak at the lowest energy became weak or null. This may be due to the photoelectrons’ inability to escape the outermost surface layer. In contrast, the pristine fullerenol sample exhibited an energy shift, most likely due to the presence of highly oxidized species associated with the hydroxyl binding onto conjugated pi bonding systems. A modified fullerenol energy sub-peak was identified at 289 eV, which can be also attributed to highly oxidized bonds such as O-C-OO (marine blue) (Nurzynska et al., 2022). The presence of these bonds may result from the chemisorption of oxygen molecules onto C-O- radicals arising from the cleavage of C=C bonds and/or from C-O- bonds present in the activated hydroxyls before silanization. Crucially, BUF-II-PEG_12_-F and OmpA-F nanobioconjugates exhibited a clearly differentiated C1s high-resolution spectra, confirming the successful conjugation of BUF-II and OmpA. The decreasing C-C/(C-O/C-N) ratio upon conjugation provided further evidence of the superior conjugation efficiency of BUF-II.

**TABLE 2:** Binding energy (BE) of the different XPS lines for C1s, O1s, N1s and S2p peak components from Fullerenol w/o silanization, F, BUF-II-F and OmpA-F nanobioconjugates samples.

Sample	C1s (BE- eV)	O1s (BE- eV)	N1s (BE- eV)	Si2p (BE- eV)
Fullerenol w/o silanization	283.3	529.8	—	—
	284.6	531.1	—	—
	285.9	532.2	—	—
	287.5	533.2	—	—
Silanized fullerenol (F)	282.9	530.7	397.3	101.6
	284.6	531.7	400.0	102.6
	287.1	532.8	402.3	103.4
	289.6	533.9	—	104.3
BUF-II-F	282.6	531.0	397.6	102.0
	284.6	532.2	400.1	102.9
	285.8	533.1	402.5	103.6
	287.5	534.1	—	104.5
	289.8	—	—	—
OmpA-F	284.6	531.1	396.7	101.3
	285.7	532.3	400.0	102.2
	287.3	533.2	402.2	103.1
	289.5	534.,2	—	104.1

Concerning the O1s-core level, a sharper peak with a slight shift to higher energy was observed for silanized and nanobioconjugates samples compared to the reference. The larger full-width of half-maximum (FWHM) value of the pristine sample is likely associated with the overall electric field’s spread on the outer C_60_-cage surface due to defects that are absent on functionalized samples. This permits the favored ejection of O1s-photoelectron at lower kinetic energies. No evidence of work function alteration due to surface charge artifacts was found since the C1s-main peak of the cage was located at 284.6 eV. Nevertheless, the C1s-peak of the reference was also broader, without any evidence of a change in the peak asymmetry compared to the functionalized samples. In contrast, the main three N1s-subpeak components were detected for silanized and nanobioconjugates samples, with a higher intensity detected for the former due to the protonated amine species of the covalently attached APTES molecules. This contrasts with the observations for the SNPs discussed above. The area under the curve for the nitrogen binding energy was lower for the nanobioconjugates compared to the silanized samples due to the lower C/N ratio after conjugation of the peptide and protein molecules. Finally, the main Si2p-subpeak components provided further evidence of the successful silanization of fullerenol.

### 3.3 Biocompatibility

Biocompatibility is a crucial property in the development of nanocarriers for biomedical applications. A material is considered biocompatible if it does not elicit an undesired response from the organism. Therefore, the assessment of biocompatibility is a fundamental step in the design and development of nanocarriers for drug delivery and diagnostic purposes. It is imperative to ensure that the nanocarriers are not toxic to the body and do not cause any adverse reactions (Soares et al., 2018). In order to ensure the biocompatibility of nanomaterials, multiple tests are required as per established standards such as the ISO 10993 series and ASTM F1903. Hemocompatibility and cytotoxicity tests are among the several necessary evaluations. The hemolytic properties, effects on blood coagulation, and cytotoxicity of the tested samples were assessed *in vitro*.

**FIGURE 8:**
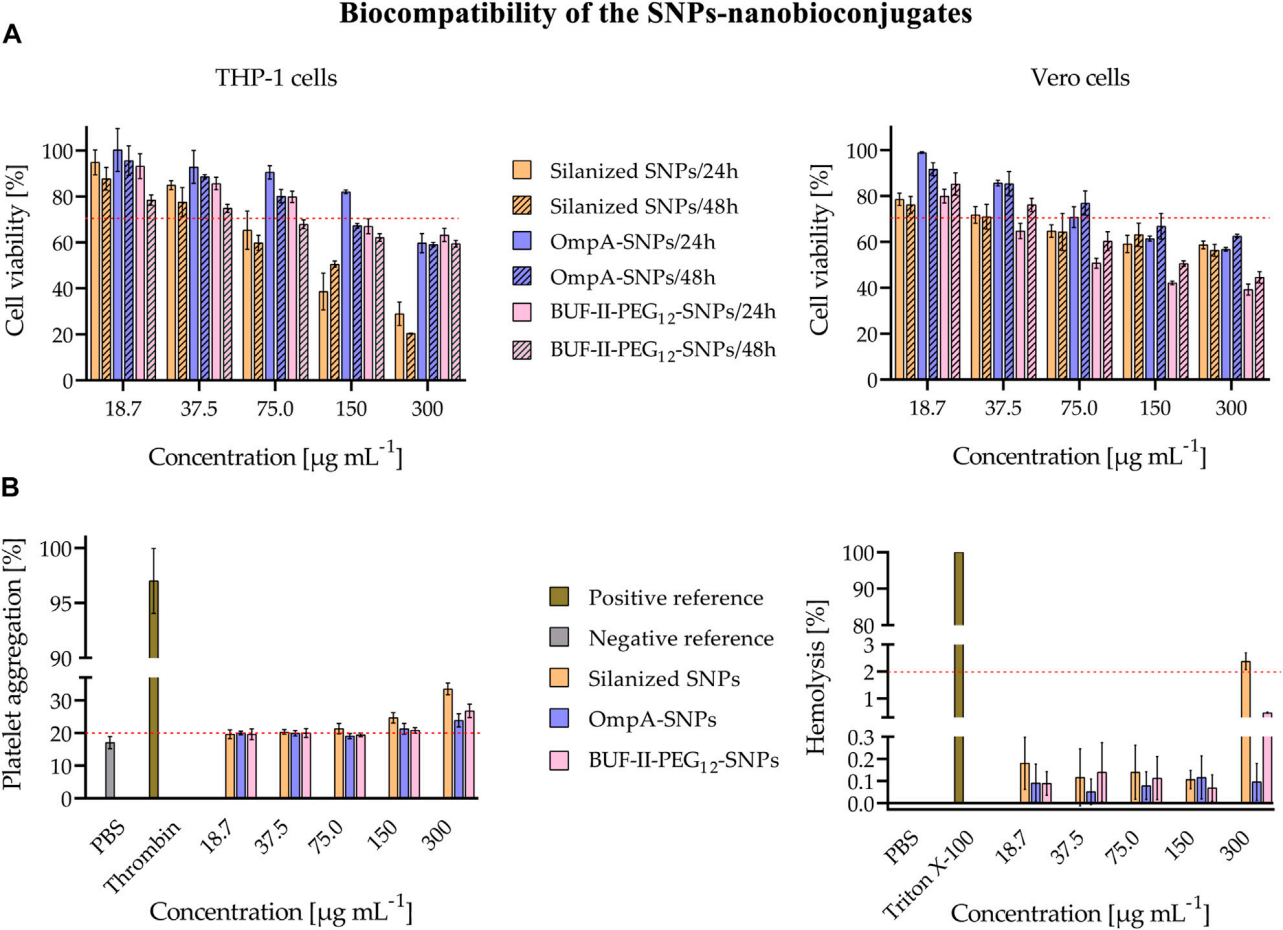
Evaluation of biocompatibility of the SNPs-nanobioconjugates **(A)** Evaluation of cytotoxicity of nanobioconjugates as tested by MTT assays after 24 and 48 h **(B)** *In vitro* evaluation of the hemocompatibility. Assessment of the hemolytic effect of nanobioconjugates (Positive control: Triton X-100, negative control: PBS. In all cases, hemolysis was below 3%; thus, the nanobioconjugates are not hemolytic; and Assessment of nanobioconjugates effects on blood coagulation (Positive control: Thrombin, negative control: PBS). There is no significant percent platelet aggregation induced by the nanobioconjugates.

As shown in Figure 8A, cell viability was evaluated in THP-1 cells—a human leukemia monocytic cell line—and Vero cells—a monkey kidney epithelial line—after 24 and 48 h of exposure to the SNPs-based treatments. The outcomes demonstrated a concentration-dependent decrease in cell viability for all treatments and cell lines. Notably, at low doses of 18 and 37 μg/mL, no significant reduction in cell viability was observed for either cell type, implying the treatments’ safety profile at lower concentrations. Moreover, it was observed that the cytotoxic potential of OmpA-SNPs was comparatively lower than that of BUF-II-PEG_12_-SNPs. The viability of cells treated with OmpA-SNPs nanobioconjugates remained above 70% (dotted line) even at concentrations as high as 75 μg mL-1. Conversely, at the same concentration, the viability of THP-1 and Vero cell lines treated with BUF-II-PEG_12_-SNPs nanobioconjugates was found to decrease. (International Organization for Standardization., 2009). In all cases, the covalent conjugation of BUF-II peptide and OmpA protein to these nanostructured materials involves the use of surface spacers (APTES, amine-PEG_12_-propionic acid, glutaraldehyde, EDC, NHS), which has rendered the nanobioconjugates less cytotoxic than the bare SNPs. Surface functionalization modified the properties of the nanoparticles—e.g., the Z-average size, the *ζ* potential, the roughness—and thus the interactions between nanoparticles and biological components, such as proteins and cell membranes, ultimately reducing cytotoxicity (Kim et al., 2013). Figure 8B shows *in vitro* evaluation of the hemocompatibility. The treatments revealed platelet aggregation values between 2% and 16% above the negative reference—values higher than 20% are considered that induce platelet aggregation (dotted line) (Potter et al., 2018). The silanized SNPs or nanobioconjugates induced no significant hemolytic effect, and the hemolysis values remained below 3% — Percent hemolysis less than 2 means the test sample is not hemolytic (dotted line); 2%–5% hemolysis means the test sample is slightly hemolytic; and >5% hemolysis means the test sample is hemolytic (Neun et al., 2018).

**FIGURE 9:**
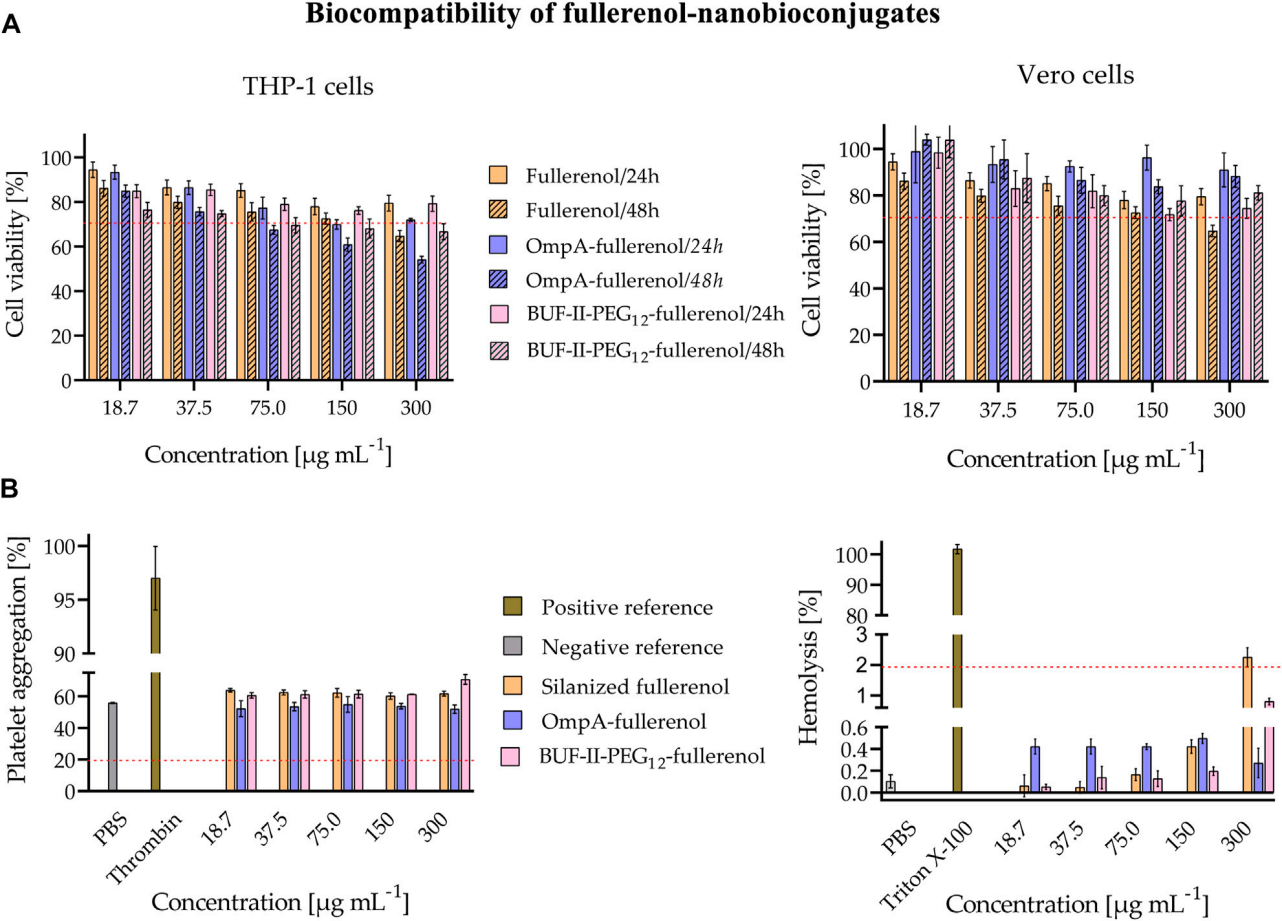
Evaluation of biocompatibility of the fullerenol-nanobioconjugates **(A)** Evaluation of cytotoxicity of nanobioconjugates as tested by MTT assays after 24 and 48 h **(B)** *In vitro* evaluation of the hemocompatibility. Assessment of the hemolytic effect of nanobioconjugates (Positive control: Triton X-100, negative control: PBS. In all cases, hemolysis was below 3%; thus, the nanobioconjugates are not hemolytic, and Assessment of nanobioconjugates effects on blood coagulation (Positive control: Thrombin, negative control: PBS). There is no significant platelet aggregation induced by the nanobioconjugates.

Similarly, in the case of nanobioconjugates that rely on fullerenol, cell viability remained uncompromised in both cell types, even at low doses of 18 and 37 μg mL-1 across all treatments. There was no significant reduction observed in the viability of the cells (Figure 9A). Figure 9B shows *in vitro* evaluation of the hemocompatibility. The results of the treatments showed platelet aggregation values that were 2%–15% higher than the negative reference in the fullerenol and BUF-II-PEG_12_-F treatment. Conversely, there was no significant difference in platelet aggregation between the negative control and the OmpA-F nanobioconjugates treatment (Potter et al., 2018). The silanized fullerenol or nanobioconjugates did not induce a significant hemolytic effect, as evidenced by the hemolysis values remaining below 3% (Neun et al., 2018).

We recognize that the dosages used in our work may not exhibit toxicity towards target cells, such as cancer or infected cells, and that altering the dosage might change the mechanism of cellular uptake. As such, future studies should investigate the potential biological actions of these nanobioconjugates using a drug model to better understand their efficacy and safety in diverse cellular contexts. This will involve characterizing how the nanobioconjugates and their cargoes are trafficked inside cells and determining the appropriate cargo release concentrations for specific cell lines. Our current research serves as a foundation for developing conjugation strategies with known cell penetration agents and for exploring the potential of silica nanoparticles and fullerenol as nanostructured supports in targeted drug delivery applications.

### 3.4 Cellular uptake and endosomal escape

**FIGURE 10:**
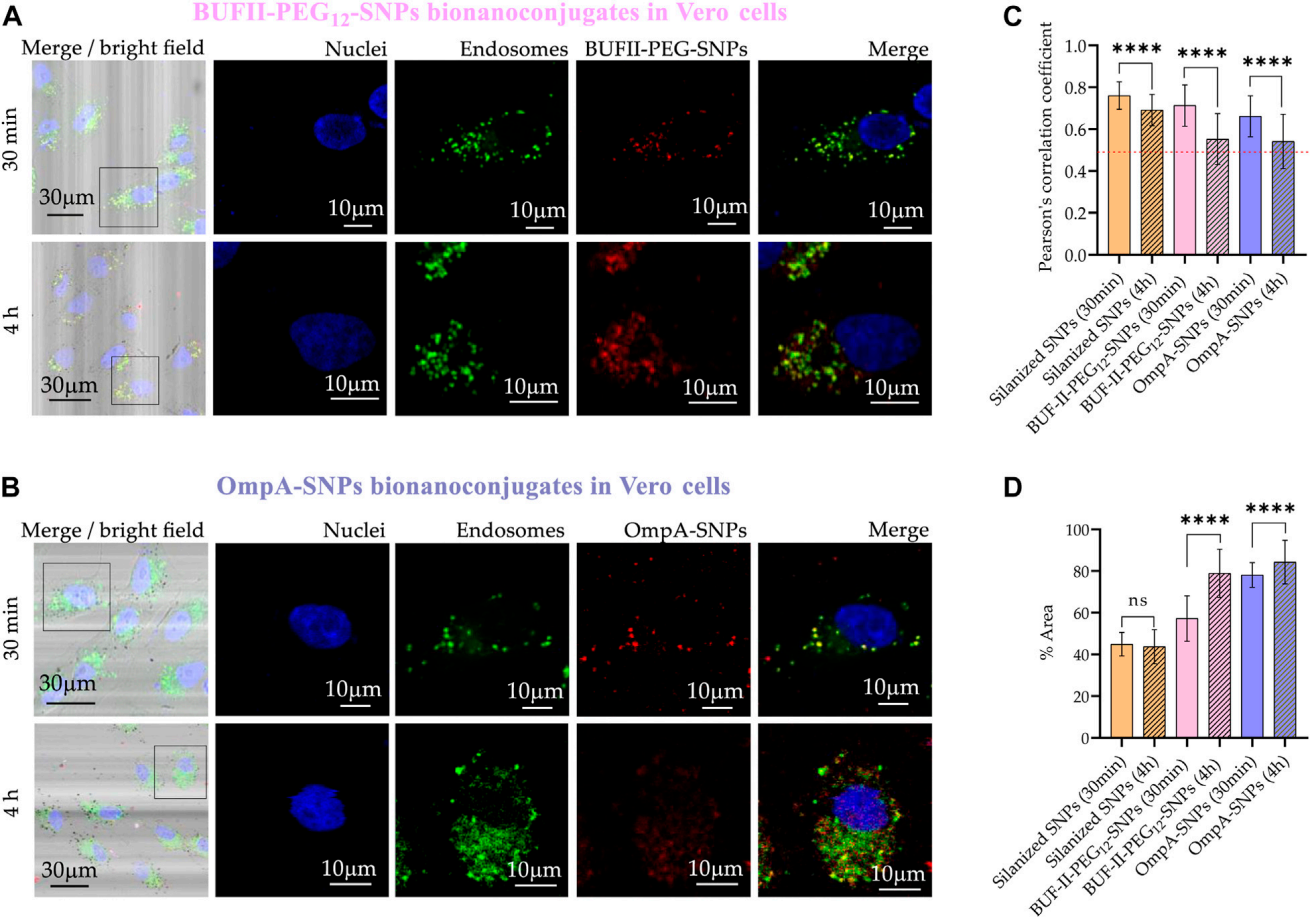
Cellular uptake and endosomal escape of the SNPs-nanobioconjugates in Vero cells **(A)** Confocal microscopy images of effective cellular uptake of BUF-II-PEG_12_-SNPs nanobioconjugates in Vero cells. A zoomed view of the insets is shown on the right **(B)** Confocal microscopy images of effective cellular uptake of OmpA-SNPs nanobioconjugates in Vero cells. A zoomed view of the insets is shown on the right **(C)** Endosomal escape study via colocalization analysis **(D)** Intracellular area percentage coverage by silanized SNPs and the nanobioconjugates for Vero cells after the two exposure times (30 min and 4 h).

Figure 10 shows the cellular uptake of silanized SNPs, OmpA-SNPs, and BUF-II-PEG_12_-SNPs nanobioconjugates by Vero cells. Rhodamine B-labeled nanobioconjugates (red) were observed to be homogeneously distributed within the cells, without significant penetration of the cell nucleus (blue). The colocalization of the nanobioconjugates with acidic organelles, such as endosomes/lysosomes, was determined quantitatively through correlation analysis, based on Pearson’s coefficient (PC). The PC value ranges from 1 to −1, where 1 represents complete and positive correlation between the intensity of fluorescence signals, −1 denotes perfect but negative correlation, and 0 indicates no correlation (Adler and Parmryd, 2010; Dunn et al., 2011). Intracellular area percentage coverage by silanized SNPs and the nanobioconjugates was also determined.

CP values after 30 min in Vero cells were 0.76 ± 0.07; 0.71 ± 0.10; and 0.66 ± 0.10 for the silanized SNPs, BUF-II-PEG_12_-SNPs, and OmpA-SNPs, respectively. These CP values decreased to 0.72 ± 0.09; 0.55 ± 0.12; and 0.54 ± 0.13; for the silanized SNPs, BUF-II-PEG_12_-SNPs, and OmpA-SNPs, respectively, after 4 h of incubation. A reduced level of colocalization with the endosomal/lysosomal marker (green) is indicative of the propensity of nanobioconjugates to evade endosomes in Vero cells. While the precise mechanisms involved in endosomal escape are not fully understood, it is likely that this occurs either through the formation of temporary pores or via the proton sponge effect, as posited in prior studies (Cho et al., 2009; Cardoso et al., 2019; López-Barbosa et al., 2019; Lopez-Barbosa et al., 2020).

**FIGURE 11:**
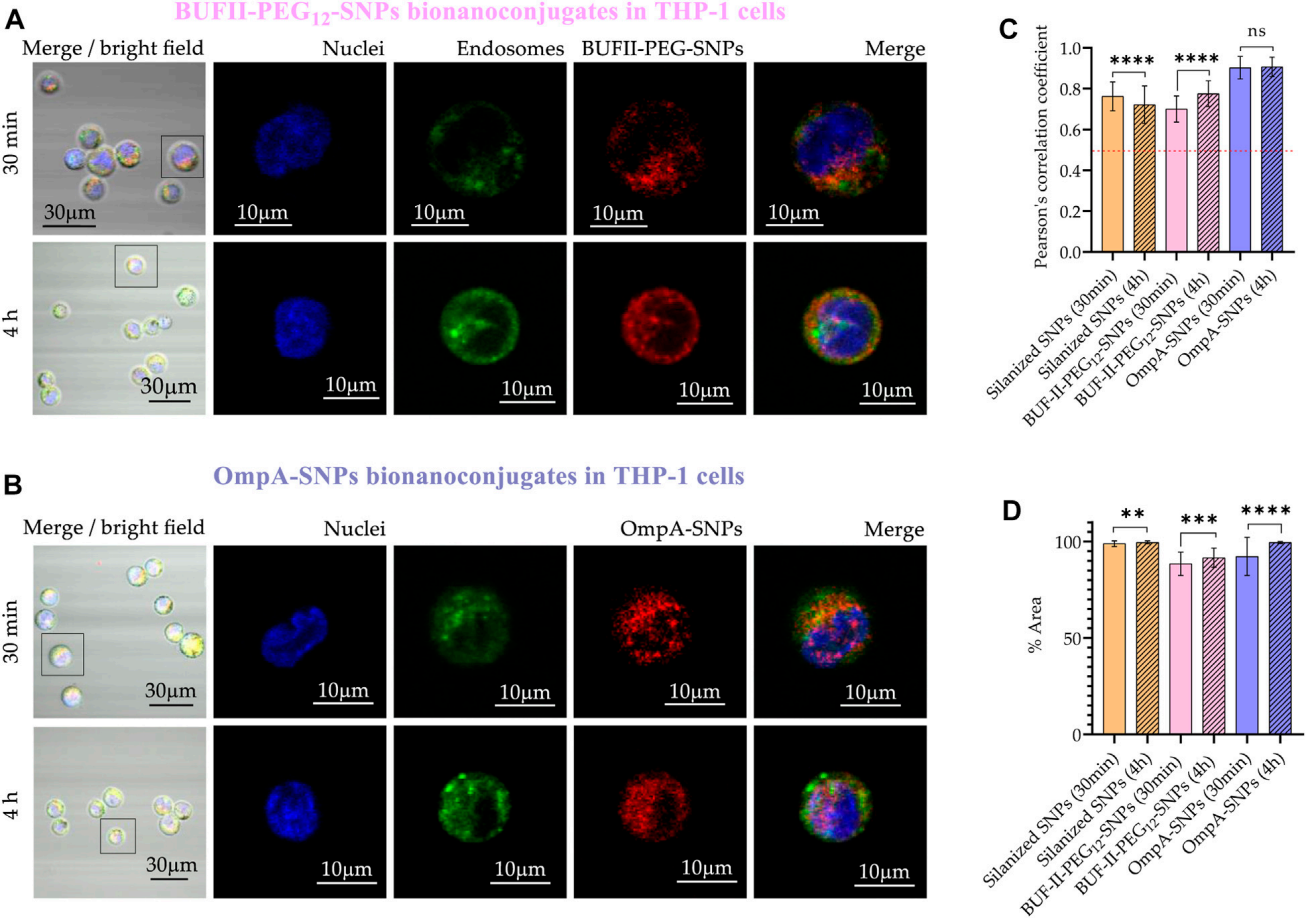
Cellular uptake and endosomal escape of the SNPs-nanobioconjugates in THP-1 cells **(A)** Confocal microscopy images of effective cellular uptake of silanized SNPs and BUF-II-PEG_12_-SNPs nanobioconjugates in THP-1 cells. A zoomed view of individual cells is shown on the insets in the right panels **(B)** Confocal microscopy images of effective cellular uptake of silanized SNPs and OmpA-SNPs in THP-1 cells. A zoomed view of individual cells is shown on the insets in the right panels **(C)** Endosomal escape study via colocalization analysis **(D)** Intracellular area percentage coverage by silanized SNPs and the nanobioconjugates for THP-1 cells after the two exposure times (30 min and 4 h).

In the case of THP-1 cells (Figure 11), CP values after 30 min were 0.76 ± 0.07; 0.70 ± 0.06; and 0.90 ± 0.05 for silanized SNPs, BUF-II-PEG_12_-SNPs, and OmpA-SNPs, respectively. After 4 h of incubation, the CP values approached 0.69 ± 0.77; 0.78 ± 0.06; and 0.91 ± 0.05; for the silanized SNPs, BUF-II-PEG_12_-SNPs, and OmpA-SNPs, respectively, indicating a low tendency of nanobioconjugates to escape from endosomes in THP-1 cells. This behavior holds potential significance for the investigation of enzyme replacement therapies in the management of lysosomal storage diseases. Such diseases necessitate periodic intravenous infusions of human recombinant lysosomal enzymes, produced through recombinant DNA techniques. Following administration of the treatment, the recombinant enzymes disperse throughout the tissues, undergo internalization by cells, and are directed to the lysosomal compartment for the purpose of substituting the deficient protein in the patients (Parenti et al., 2013). Finally, the percentages of the area covered by the nanobioconjugates were higher in THP-1 cells than in Vero cells and increased with incubation time.

**FIGURE 12:**
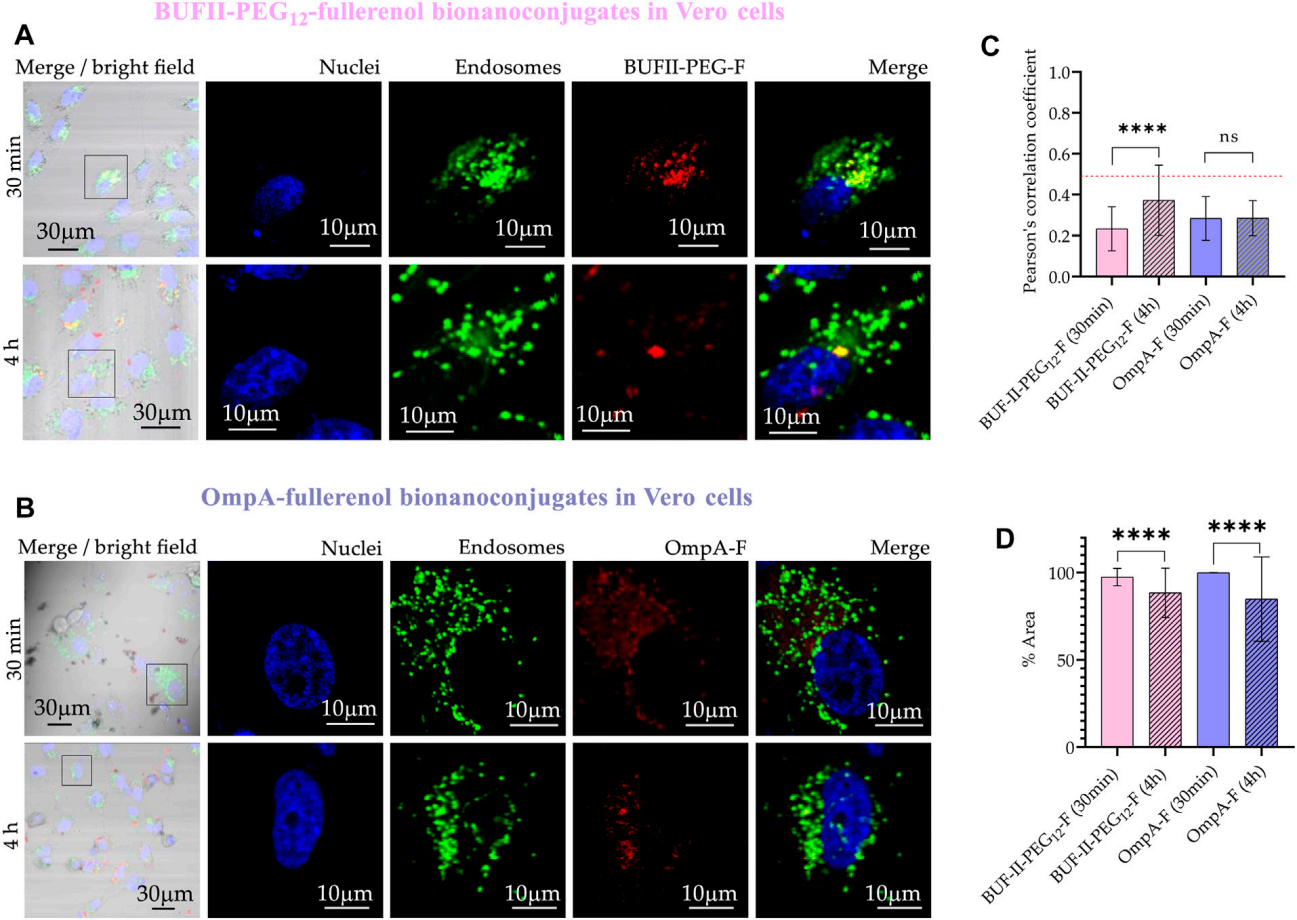
Cellular uptake and endosomal escape of the fullerenol-nanobioconjugates in Vero cells **(A)** Confocal microscopy images of effective cellular uptake of BUF-II-PEG_12_-Fnanobioconjugates in Vero cells. A zoomed view of individual cells is shown on the insets in the right panels **(B)** Confocal microscopy images of effective cellular uptake of OmpA-F nanobioconjugates in Vero cells. A zoomed view of individual cells is shown on the insets in the right panels **(C)** Endosomal escape study via colocalization analysis **(D)** Intracellular area percentage coverage by the nanobioconjugates for Vero cells after the two exposure times (30 min and 4 h).

Figure 12 provides evidence of the cellular uptake of OmpA-F and BUF-II-PEG_12_-F nanobioconjugates by Vero cells. The rhodamine B-labeled nanobioconjugates (red) were observed to be homogeneously distributed within the cells but did not significantly reach the cell nucleus (blue). In contrast to SNP-based nanobioconjugates, fullerenol-based nanobioconjugates tend to aggregate and form clusters, which could have significant implications for their biological applications. CP values after 30 min were 0.23 ± 0.11; and 0.28 ± 0.11 for BUF-II-PEG_12_-F and OmpA-F, respectively. After 4 h of incubation, the CP values approached 0.37 ± 0.17; and 0.29 ± 0.08; for BUF-II-PEG_12_-F and OmpA-F, respectively. CP values less than 0.5 indicate a low degree of colocalization between the nanobioconjugates and the endosomes/lysosomes. The extensive coverage of the cytoplasmic area by the particles provides evidence for the internalization of the nanobioconjugates. These results suggest that the mechanism of entry of the nanobioconjugates into Vero cells is likely non-endocytic.

**FIGURE 13:**
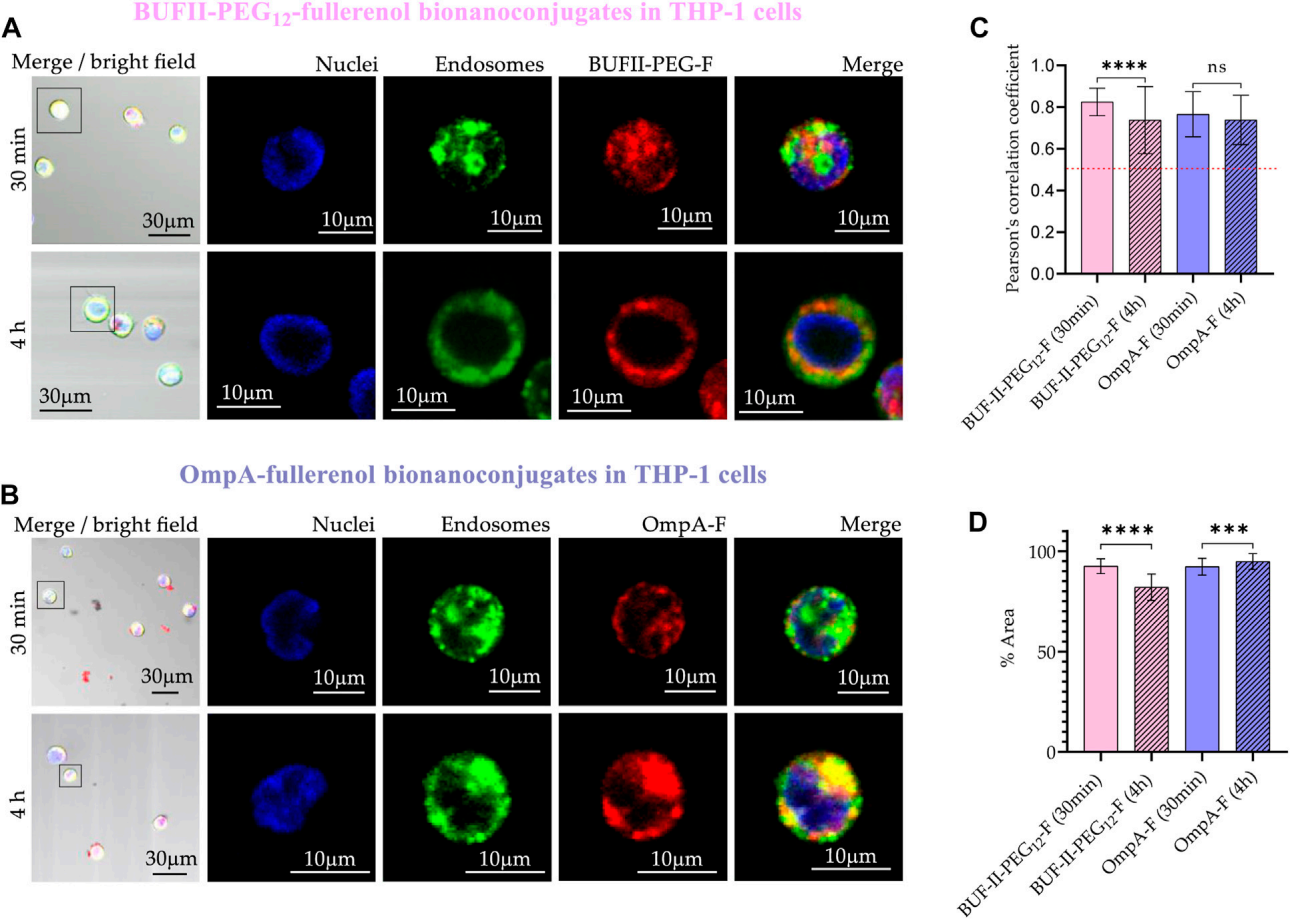
Cellular uptake and endosomal escape of the fullerenol-nanobioconjugates in THP-1 cells **(A)** Confocal microscopy images of effective cellular uptake of BUF-II-PEG_12_-F nanobioconjugates in THP-1 cells. A zoomed view of individual cells is shown on the insets in the right panels **(B)** Confocal microscopy images of effective cellular uptake of OmpA-F nanobioconjugates in THP-1 cells. A zoomed view of individual cells is shown on the insets in the right panels **(C)** Endosomal escape study via colocalization analysis **(D)** Intracellular area percentage coverage by the nanobioconjugates for THP-1 cells after the two exposure times (30 min and 4 h).

In the case of THP-1 cells (Figure 13), CP values after 30 min were 0.83 ± 0.07; and 0.77 ± 0.11 for BUF-II-PEG_12_-F and OmpA-F, respectively. These CP values decreased to 0.74 ± 0.16; and 0.74 ± 0.12; for BUF-II-PEG_12_-F and OmpA-fullerenol, respectively, after 4 h of incubation. This suggests a tendency of BUF-II-PEG_12_-F nanobioconjugates to escape from endosomes in THP-1 cells, which is not observed for OmpA-F nanobioconjugates. The high coverage of the cytoplasmic area confirms the internalization of the nanobioconjugates.

In addition to the cell penetration and endosomal escape capabilities of our nanobioconjugates, it is essential to highlight their potential for targeted drug delivery to specific subcellular compartments. By functionalizing the nanobioconjugates with appropriate ligands, such as specific peptide sequences or small molecules, these drug delivery systems can be tailored to exhibit a high affinity for the desired organelle. This customization enables enhanced specificity in targeting organelles such as mitochondria or others of interest. Moreover, we acknowledge the potential benefits of non-endocytic mechanisms as a more straightforward route for targeting subcellular compartments. While endosomal escape is a crucial step in ensuring efficient delivery of cargo to the cytosol, non-endocytic routes might offer alternative advantages in achieving more targeted delivery to specific organelles. Further exploration of these strategies and the development of suitable ligands will be essential in optimizing the nanobioconjugates for specific therapeutic applications.

## 4 Conclusion and outlook

In summary, our study demonstrates a comprehensive approach for immobilizing translocating biomolecules on SNPs and fullerenol. The success of this strategy was confirmed by a range of analytical techniques including FT-IR, TGA, DLS, Electrophoretic Mobility, SEM, TEM, and XPS. The resulting nanobioconjugates, including OmpA-SNPs, BUF-II-PEG_12_-SNPs, OmpA-F, and BUF-II-PEG_12_-F, exhibited high biocompatibility in both Vero and THP-1 cell lines. Moreover, our evaluations of hemolytic effects and platelet aggregation demonstrated their safety at the tested concentrations. Our confocal microscopy studies revealed efficient internalization of the different nanobioconjugates in both Vero and THP-1 cells, with notable differences in endosomal escape. In particular, OmpA-SNPs and BUF-II-PEG_12_-SNPs showed a tendency to escape from endosomes in Vero cells, while remaining trapped in THP-1 cells. On the other hand, OmpA-F and BUF-II-PEG_12_-F were effectively internalized by both cell lines, with a superior tendency to escape from endosomes in Vero cells. These findings are significant, as they provide evidence for the potential of our nanobioconjugates to enhance the stability and half-life of translocating biomolecules and cross biological membranes without affecting cell viability. The ability to develop highly tunable cargo delivery systems is crucial for meeting the needs of specific treatments and targeting cell or organelle types. Overall, our study highlights the promise of our nanobioconjugates as a platform for the development of innovative therapeutic approaches. In our forthcoming research, we aim to elucidate the intracellular trafficking mechanisms of these nanobioconjugates and their cargoes, as well as the targeted release of cargo within specific cell lines at precise concentrations.

## Data Availability

The original contributions presented in the study are included in the article/Supplementary Material, further inquiries can be directed to the corresponding author.
