# Supplementary material for: Unlocking cellular barriers: silica nanoparticles and fullerenol conjugated cell-penetrating agents for enhanced intracellular drug delivery
Source: Front Bioeng Biotechnol. 2023 May 9;11:1184973. doi: 10.3389/fbioe.2023.1184973 (PMC10203439; doi:10.3389/fbioe.2023.1184973)
Supplement: Supplementary file 1 [file DataSheet1.docx]

Supplementary Material

Unlocking Cellular Barriers: Silica Nanoparticles and Fullerenol Conjugated Cell-Penetrating Agents for Enhanced Intracellular Drug Delivery

Eduardo Ravelo-Nieto ^1,2^, Javier Cifuentes ^2^, Paola Ruiz Puentes ^2^ , Laura Rueda-Gensini ^2^, Valentina Quezada ^2^, Carlos Ostos ^3^, Carolina Muñoz-Camargo ^2^, Luis H. Reyes ^4^, Alvaro Duarte-Ruiz ^1^, Juan C. Cruz ^2,*^

^1^Department of Chemistry, Universidad Nacional de Colombia, Ciudad Universitaria, Bogotá Cra. 30 No 45-03, Colombia; eravelo@unal.edu.co (E.R.-N); aduarter@unal.edu.co (A.D.-R)

^2^Department of Biomedical Engineering, Universidad de los Andes, Cra. 1E No. 19a – 40, Bogotá, DC 111711, Colombia; jf.cifuentes10@uniandes.edu.co (J.C.); p.ruiz@uniandes.edu.co (P.R.P); l.ruedag@uniandes.edu.co (L.R.G); v.quezada@uniandes.edu.co (V.Q); jc.cruz@uniandes.edu.co (J.C.C)

^3^Grupo CATALAD, Instituto de Química, Universidad de Antioquia, Medellín 050010, Colombia; carlos.ostos@udea.edu.co (C.O.)

^4^Grupo de Diseño de Productos y Procesos (GDPP), Department of Chemical and Food Engineering, Universidad de los Andes, Cra. 1E No. 19a – 40, Bogota DC 111711, Colombia; [lh.reyes@uniandes.edu.co](mailto:lh.reyes@uniandes.edu.co) (LH.R.)

*** Correspondence:**

Juan C. Cruz
jc.cruz@uniandes.edu.co; Tel.: +57-1-3394949 (ext. 1789)

# Supplementary Data

1. BUF-II and OmpA bioconjugation 100 mg of SNPs were used:

$$Area SNPs \left( A_{SNPs} \right)=4\pi r^{2}$$

$$\left( A_{SNPs} \right)=4\pi{(\frac{1.76x{10}^{-4}mm}{2})}^{2}=9.73x{10}^{-8}{mm}^{2}=9.73x{10}^{13}{nm}^{2}$$

$$Volumen SNPs \left( V_{SNPs} \right)=\frac{4}{3}\pi r^{3}$$

$$\left( V_{SNPs} \right)=\frac{4}{3}\pi({\frac{1.76x{10}^{-4}mm}{2})}^{3}=2.85x{10}^{-12}{mm}^{3}$$

APTES amount calculations (spherical molecule is assumed):

Number of molecules ($N_{molecules})$ of APTES in one particle:

$$N_{molecules}=\frac{A_{SNPs}}{sa APTES}$$

$$N_{molecules}=\frac{9.73x{10}^{-8}{mm}^{2}}{{5.37x10}^{-13} {mm}^{2}}=181210 APTES molecules$$

According to theoretical calculations, there will be 181210 NH_2_ groups available for functionalization.

Number of particles 100 mg $(N_{particles})$:

$$N_{SNPs}=\frac{0.100 g}{W_{SNPs}}=\frac{0.100 g}{{6.85x10}^{-15}g}={1.46x10}^{13}SNPs$$

Number of necessary Mol ($N_{mol})$APTES per particle:

$$N_{mol}=\frac{N_{SNPs}*N_{molecules} APTES}{{6.02x10}^{23} molecules/mol}$$

$$N_{mol}=\frac{{1.46x10}^{13}SNPs*181210 APTES molecules}{{6.02x10}^{23} molecules/mol}={4.39x10}^{-6}APTES molecules$$

After including the excess of APTES, and glutaraldehyde the same amount of COOH sites of the carboxyl end NH_2_-PEG_12_-prop. acid that is the number of molecules of peptides and proteins that need to be added to saturate the active functionalization sites.

$$W_{BUF-II}={4.39x10}^{-6}BUF mol-II*MW BUF-II$$

$$W_{BUF-II}={4.39x10}^{-6}BUF mol-II*2434,8\frac{g}{mol}=0.0107 g$$

$$W_{BUF-II}=0.0107 g*\frac{100\%}{99\%}=0.0108 g BUF-II=10.8 mg de BUF-II$$

$$W_{OmpA}={4.39x10}^{-6}OmpA mol*MW OmpA$$

$$W_{OmpA}={4.39x10}^{-6}OmpA mol*35000\frac{g}{mol}=0.1538 g$$

$$W_{OmpA}=0.1538 g*\frac{100\%}{94\%}=0.163 g OmpA=163.6 mg OmpA$$

Due to cost efficiency and because the objective was not to saturate all available sites, in this bioconjugation reaction of OmpA to SNPs, only 1 mg of BUF-II and 30 mg of OmpA were used for every 100 mg of SNPs.

**Supplementary Figure 1.** Fullerenol-PEG_12_-BUF-II nanobioconjugation, via a multi-step reaction using glutaraldehyde, amine-PEG_12_-propionic acid, and EDC/NHS to form an amide bond between a carboxylate and N-terminal region of the peptide BUF-II.

**Supplementary Figure 2.** Fullerenol-OmpA nanobioconjugation, using glutaraldehyde as the crosslinking agent.
